# RUNX1 promotes angiogenesis in colorectal cancer by regulating the crosstalk between tumor cells and tumor associated macrophages

**DOI:** 10.1186/s40364-024-00573-1

**Published:** 2024-02-28

**Authors:** Xuxue Guo, Haonan Zhang, Chengcheng He, Kaiwen Qin, Qiuhua Lai, Yuxin Fang, Qianhui Chen, Weize Li, Yiqing Wang, Xinke Wang, Aimin Li, Side Liu, Qingyuan Li

**Affiliations:** 1 Guangdong Provincial Key Laboratory of Gastroenterology, Department of Gastroenterology, Nanfang Hospital, Southern Medical University, No. 1838, Guangzhou Avenue North, Guangzhou, 510515 People’s Republic of China; 2 Department of Gastroenterology, the Second Affiliated Hospital of Guangzhou Medical University, Guangzhou, Guangdong China; 3 Department of Gastroenterology, The Third Affiliated Hospital of Guangzhou Medical University, Guangzhou, Guangdong China; 4 The First School of Clinical Medicine), Nanfang Hospital, Southern Medical University, Guangzhou, Guangdong China; 5 State Key Laboratory of Organ Failure Research, Guangdong Provincial Key Laboratory of Viral Hepatitis Research, Department of Hepatology Unit and Infectious Diseases, Nanfang Hospital, Southern Medical University, Guangzhou, Guangdong China; 6 Department of Pathology, Nanfang Hospital, Southern Medical University, Guangzhou, Guangdong China; 7 Department of Pathology, School of Basic Medical Sciences, Southern Medical University, Guangzhou, Guangdong China; 8 Pazhou Lab, Guangzhou, Guangdong China

**Keywords:** Angiogenesis, Colorectal cancer, RUNX1, M2 polarization, Tumor associated macrophages

## Abstract

Colorectal cancer (CRC) is a common malignancy worldwide. Angiogenesis and metastasis are the critical hallmarks of malignant tumor. Runt-related transcription factor 1 (RUNX1), an efficient transcription factor, facilitates CRC proliferation, metastasis and chemotherapy resistance. We aimed to investigate the RUNX1 mediated crosstalk between tumor cells and M2 polarized tumor associated macrophages (TAMs) in CRC, as well as its relationship with neoplastic angiogenesis. We found that RUNX1 recruited macrophages and induced M2 polarized TAMs in CRC by promoting the production of chemokine 2 (CCL2) and the activation of Hedgehog pathway. In addition, we found that the M2 macrophage-specific generated cytokine, platelet-derived growth factor (PDGF)-BB, promoted vessel formation both *in vitro* and *vivo*. PDGF-BB was also found to enhance the expression of RUNX1 in CRC cell lines, and promote its migration and invasion *in vitro*. A positive feedback loop of RUNX1 and PDGF-BB was thus formed. In conclusion, our data suggest that RUNX1 promotes CRC angiogenesis by regulating M2 macrophages during the complex crosstalk between tumor cells and TAMs. This observation provides a potential combined therapy strategy targeting RUNX1 and TAMs-related PDGF-BB in CRC.

**Supplementary Information:**

The online version contains supplementary material available at 10.1186/s40364-024-00573-1.

## Introduction

Colorectal cancer (CRC) is one of the most common malignant tumors worldwide with a 10.0% incidence and 9.4% mortality ranking third and second, respectively [1]. Tumor angiogenesis plays a crucial role in promoting the growth and metastasis of human malignancies, including CRC [2, 3]. A growing body of evidence indicates that blocking blood supply can effectively inhibit primary tumors and diminish the frequency and extent of metastasis [4]. Unfortunately, only a minority of CRC patients respond well to anti-angiogenic therapy [5], and the reasons for this are currently an area of intense research. Hence, new therapeutic strategies to enhance antitumor efficacy are urgently needed.

Runt-related transcription factor 1 (RUNX1), a member of the RUNX family (RUNX1, RUNX2 and RUNX3), controls hematopoiesis and angiogenesis in vertebrates [6]. It is indispensable for the embryogenesis and bone marrow blood stem cell differentiation [6]. Interestingly, this transcription factor also functions as a tumor suppressor or an oncogene in a variety of solid tumors [7–11]. In recent years, the carcinogenic effect of RUNX1 has attracted increasing attention in the field of cancer, including non-small-cell lung cancer, renal cell carcinoma, ER-negative breast cancer, endometrial cancer, etc [9–11]. Our previous work suggested that RUNX1 promotes CRC metastasis by activating the Wnt/β-catenin signaling pathway and epithelial-mesenchymal transition [12]. In addition, RUNX1 acts as an oncogene via the Hedgehog signaling pathway activation to facilitate CRC cell proliferation [13]. In particular, our previous studies illustrated that RUNX1 reduces the sensitivity of CRC chemotherapy by up-regulating the expression of ABCG2 [13]. Nevertheless, to this day the precise mechanism guiding the RUNX1 modulated tumor angiogenesis remains unknown.

The connections between tumor cells and their surrounding tumor microenvironment (TME) are known to be crucial for malignant progression [14]. Tumor associated macrophages (TAMs), the most abundant cells residing in the solid TME, are heterogeneous and display considerable plasticity [15]. Those immune cells can generally be classified into M1 (classically activated) macrophages and M2 (alternatively activated) macrophages according to their polarization state [15]. M2 subtype TAMs are typically polarized in the presence of M-CSF, IL-4, IL-10 and IL-13, and oriented toward remodeling extracellular matrix, promoting angiogenesis, immune evasion, and promoting tumor growth and metastasis [16–23], while the M1 phenotype is induced under the influence of IFN-γ (interferon-γ), GM-CSF (granulocyte macrophage colony stimulating factor) and TLR (Toll-like receptor) agonists [24], and reportedly antitumoral and linked with better prognosis in CRC patients [25]. High levels of M2 macrophages infiltration have been reported to be associated with poor prognosis in patients with CRC [26, 27]. Although the treatment targeting CD163^+^ TAMs promotes tumor regression, the specific interaction mode of TAMs and tumor cells is unclear [28].

Neither the complex crosstalk between RUNX and TAMs nor the way in which the former increases M2 TAMs infiltration or induces angiogenesis in CRC are clear. Here, we provide the first evidence that CRC-derived RUNX1 recruits macrophages in the TME via the secretion of chemokine 2 (CCL2). In addition, we show that RUNX1 skewed macrophage differentiation toward the M2 phenotype by activating the Hedgehog pathway, and subsequently promoted M2 TAMs to produce angiogenesis related cytokine platelet-derived growth factor (PDGF)-BB. M2 TAMs derived PDGF-BB promotes tumor angiogenesis both *in vitro* and *in vivo*. Finally, we show that M2 TAMs secreted PDGF-BB stimulated RUNX1 expression in CRC cells and concurrently promoted CRC migration and invasion *in vitro*. In conclusion, these results indicate that CRC-derived RUNX1 regulates TAMs function to promote angiogenesis in the TME in CRC.

## Materials and methods

For cell culture, stable cell lines generation, cell proliferation assay, cellular wound healing assay, cell transwell migration and invasion assays, tube formation on Matrigel, qRT-PCR (quantitative real-time polymerase chain reaction) (The specific primers used are listed in Supplementary Table 1), western blotting, enzyme-linked immunosorbent assay (ELISA) and bioinformatics analysis concerning RUNX1 see Supplemental Methods.

### Clinical specimens

A total of 30 patients who underwent radical operation for CRC at Nanfang Hospital of Southern Medical University were included in the study after obtaining informed consent. A diagnosis of CRC was histopathologically confirmed for each patient sample. Thirty pairs of cancer and matched normal tissues were prepared for qRT-PCR analysis, and twelve pairs of which were randomly selected for immunohistochemistry (IHC) analysis. Additionally, none of the patients had received radiotherapy or chemotherapy before surgery. The protocols used in this paper were approved by Nanfang Hospital’s Protection of Human Subjects Committee (NFEC-201809-K3).

### Conditioned mediums (CMs) preparation and CRC cells-THP-1 co-culture

To obtain CRC cell-derived CMs, stably transfected CRC cells were seeded on 10 cm dishes for 24 h, and then supernatants were collected and filtered for follow-up usage (Fig. 3A). For CRC cells-THP-1 co-culture experiments, CRC cells were seeded in 24-mm diameter inserts with 0.4-μm pore (3412, Corning Incorporated, USA), then transferred to a 6-well plate seeded with THP-1 macrophages in advance. After being cocultured for another 24 h, CRC cells/THP-1-derived CMs was collected and filtered for follow-up usage. In the co-culture experiments, 500 ng/ml phorbol 12-myristate 13-acetate (PMA) was added beforehand for 12 h to differentiate THP-1 into adhered macrophages (M0). To prepare THP-1-derived CMs, adhered THP-1 macrophages induced by PMA were cultured for 24 h in the presence of 100 ng/ml IL-4 or PBS, then washed twice with PBS, and cultured in fresh DMEM complete medium for another 24 h. Finally, the culture medium was filtered and stored at -80 ℃ for follow-up usage.

### IHC analysis

IHC staining was employed following the manufacturer’s instructions (PV-6001, ZSGB-BIO, Beijing, China) using RUNX1 (1:100, 25315-1-AP, Proteintech), CCL2 (1:300, 25542-1-AP, Proteintech), CD68 (ZM-0060, ZSGB-BIO), CD163 (ZM-0428, ZSGB-BIO), F4/80 (1:800, 29414-1-AP, Proteintech) and CD31 (1:500, 28083-1-AP, Proteintech). Two independent pathologists used software ImageJ to calculate the proportion of positive area. Data were expressed as the average of three randomly selected microscopic fields.

### Immunofluorescence (IF) assay

For cellular IF, cells were seeded on 15 mm glass-bottom dishes. After the indicated treatment, the cells were cultured for 48 h, fixed with 4% formaldehyde for 10 min at room temperature (RT), and washed three times with wash buffer (0.02% Tween 20/PBS). Then, the cells were permeabilized with 0.5% Triton X-100/PBS for 10 min at RT. The cells were washed three times with wash buffer (5 min each time) and then incubated with 1.5% bovine serum albumin (BSA)/phosphate-buffered saline (PBS) solution (blocking solution) for 30 min at RT. The RUNX1 (25315-1-AP, Proteintech) was incubated in blocking solution at 4°C overnight. After washing, the cells were incubated with Alexa488-conjugated secondary antibodies (Proteintech, SA00006-2, 1:300 in blocking buffer) for 60 min at RT in the dark followed by counterstaining with DAPI (Thermo Fisher). For CD206 and CD200R double IF staining, THP-1 cells treated with CRC cells-derived CMs were incubated with fluorescent labeled anti-human‐CD206 (321104, BioLegend) and anti-human‐CD200R (329308, BioLegend) antibody for 30 min at RT in the dark. Finally, samples were imaged on a laser-scanning confocal microscope (× 200), and mean fluorescence intensity (Integrated Density/Area) of the IF images was calculated to semi-quantitatively characterize the expression of CD206 and CD200R via ImageJ software.

For IF labeling of mouse orthotopic tumor, paraffin sections were deparaffinized and rehydrated in advance, and then antigen retrieval and blocking were performed before staining. The CD31 (1:200, 11265-1-AP, Proteintech) or CCL2 (1:100, 25542-1-AP, Proteintech) was incubated in blocking solution at 4°C overnight. After washing, samples were incubated with Alexa488-conjugated secondary antibodies (Proteintech, SA00006-2, 1:300 in blocking buffer) or CoraLite594-conjugated Goat Anti-Rabbit IgG(H+L) (Proteintech, SA00013-4, 1:200 in blocking buffer) for 60 min at RT in the dark followed by counterstaining with DAPI (Thermo Fisher). Multi-label IF staining of tumor tissue in CRC patients was performed. Firstly, paraffin sections were deparaffinized and rehydrated in advance, and then antigen retrieval and blocking were performed before staining. The CY3-RUNX1 (1:200, 4336, CST), CY5-CD163 (1:500, ab182422, Abcam) and Alexa488-CD68 (1:1000, ab955, Abcam) was subsequently incubated in blocking solution at 4°C overnight. After washing, samples were correspondingly incubated with secondary antibodies (SeraCare, 5220-0336 or 5220-0341, 1:400 in blocking buffer) for 60 min at RT in the dark followed by counterstaining with DAPI (Thermo Fisher). Samples were imaged on an Olympus fluorescence microscope.

### Flow cytometry

To evaluate the effect of RUNX1 on M2 polarization of TAMs, macrophages were washed, trypsin digested or scraped off, and processed into single-cell suspensions after the supernatant was collected for cytokine detection. Then the cell samples were labeled with antibodies as follows: PE anti-human CD68 (333808, BioLegend), FITC anti-human‐CD206 (321104, BioLegend) and APC anti-human‐CD200R (329308, BioLegend). After membrane staining of CD206 and CD200R, intracellular staining for CD68 was performed with Fix/Perm buffer reagents (421002, 420801, BioLegend) according to the manufacturer’s procedure. M2 macrophages were identified as CD68^+^CD206^+^ cells, CD68^+^CD200R^+^ cells. For the rescue experiments, THP-1 was stimulated with CMs derived from HCT116 NC/RUNX1^OE^ cells along with or without GDC-0449. The cell samples were stained with the above protocol. The cells were measured using a FACS Calibur flow cytometer (BD FACSAria III, BD Biosciences), and the acquired data was analyzed by FlowJo software (Tree Star, San Carlos, Calif., USA).

### Dual luciferase reporter assay

HCT116-Ctrl, HCT116-RUNX1, SW480-Ctrl and SW480-RUNX1 cells of 80% confluence were transfected with indicated plasmids using Lipofectamine 3000 (Invitrogen, Carlsbad, CA, USA). A pGL4.10 vector subcloned with or without the CCL2-3′ UTR was co-transfected per well of a 24-well plate. Cell extracts were prepared at 36 h after transfection. The luciferase activity was determined by a Dual Luciferase Reporter Assay System (Promega, Madison, WI, USA).

### RNA-Seq

Total RNA from 4 groups of CRC cells with RUNX1 overexpression or depletion was subjected to Hiseq RNA-Seq, performed by BGI Tech Solutions Co., Ltd, China. Transcriptome reads from RNA-Seq experiments were mapped to the reference genome by using Bowtie tool (DOI:10.6084/m9.figshare.25126991.). Gene expression level was quantified by a software package called RSEM. The list of significance was operated by setting a *P* value threshold at a level of 0.05. The differentially expressed genes were subsequently analyzed for heat map analysis and enrichment of signaling pathways using Wikipathway Cancer platform (http://www.webgertalt.org/).

Chromatin immunoprecipitation (ChIP)-qPCR and DNA agarose gel electrophoresis

The ChIP assays were performed using a Merck ChIP Kit (#17-10085, Merck, German). The procedure was as described in the kit provided by the manufacturer. Briefly, RUNX1 over-expressed HCT116 cells (1 × 10^7^) as indicated were crosslinked with 1% formaldehyde, fragmented by sonication. Then, 5ug RUNX1 antibody (25315-1-AP, Proteintech) was used to immunoprecipitated the DNA-protein complex overnight at 4 ℃; 5M NaCl and proteinase K treatment was used to undo DNA-protein crosslinking at 65 ℃ for 2h. After washing and reverse-crosslinking, the precipitated DNA was amplified by primers and quantified by the qPCR. Fold enrichment was calculated after normalizing to 1% input. Primer sequences can be found in the Supplemental Table 1. The high quality of isolated DNA samples was amplified by PCR, and then subjected to electrophoresis in 1.2% agarose gel and visualized under ultraviolet light.

Chick embryo chorioallantoic membrane (CAM) assay

Fertilized specific-pathogen-free (SPF) chicken eggs (Xinxing dahuanong poultry egg Co., Ltd, Guangdong, China) were disinfected with 70% ethanol, and then incubated at 37°C with 60% relative humidity for embryogenesis. On the eighth day of fertilized egg incubation, a tiny round window (2-3 cm in diameter) was drilled into the eggshell right through air chamber and the eggs were sealed with sterile tape to avoid contamination of its contents. On the ninth day of embryo development, 80 μl CMs of different groups were added onto the developing CAM (3 in each group) using an 8 mm sterile plastic ring before resealing the eggs. The whole procedure was performed under aseptic conditions. Three days later (Day 12 of incubation), the CAM was cut off after being fixed in 2 ml fixing solution (methanol: acetone 1:1) for 15min, and then unfolded in distilled water, laid on filter paper and photographed. To quantify the angiogenesis, the ratio of vascular area to CAM area was evaluated via the software ImageJ.

### In vivo Matrigel plug assay

A total of 500 µL mixture consisting of 250 µL Matrigel Matrix (354248, Corning) and 250 µL CMs containing (4 × 10^6^) HUVEC was subcutaneously (s.c.) injected in the dorsal flank of the nude mice (3 in each group) forming a firm plug. After seven days the mice were sacrificed and the Matrigel plug together with surrounding granulation tissue were removed. The Matrigel plugs were fixed in formaldehyde, embedded into paraffin blocks, and sectioned into slides of 5 µm for hemogenic endothelium (H&E) and CD31 staining analysis.

### In vivo animal model experiments

Female athymic 4 to 5-week-old BALB/c nude mice were purchased from the Laboratory Animal Services Centre of Guangdong Province and were maintained in SPF facility. All experimental procedures were approved by the Animal Ethics Committee of Southern Medical University. For the orthotopic injection metastatic mouse model assay, randomly divided nude mice (3 in each group) were anesthetized with 1% pentobarbital, and (5 × 10^6^) cells belonging to different groups were injected under the ileocecal serosa. The general growth conditions in the nude mice were observed after surgery to determine whether there were signs of cachexia, such as wasting and arched backs. Then, 40 days after the operation, all mice were sacrificed, and the tumors were resected for H&E and IHC analysis. For the pulmonary metastasis mouse model of CRC assay, HCT116 cells were firstly stimulated with THP-1-derived CMs for 24h (CM^THP-1+PMA+PBS^ group, CM^THP-1+PMA+IL-4^ group), and then were inoculated into randomly divided nude mice (5 in each group, 2 × 10^6^/200ul per mouse) via caudal vein. About 4 weeks later, all mice were sacrificed, and the lungs were resected for H&E analysis.

### Statistical analysis

All statistical analyses were performed using GraphPad Prism 8.0 (GraphPad Software, San Diego, CA, USA). Pearson’s correlation coefficients were used to evaluate the relationship between CD163, Arg1, CD68, CCL2, CD206, IL-10 expression and RUNX1 expression. Groups of discrete variables were compared using the Student's t-test or one-way ANOVA. All experiments for cell cultures were performed independently at least three times and in triplicates each time. *P < 0.05, **P < 0.01, ***P < 0.001, and ****P < 0.0001 were considered significant.

## Results

### RUNX1 promotes CCL2-mediated recruitment of TAMs

**Fig. 1:**
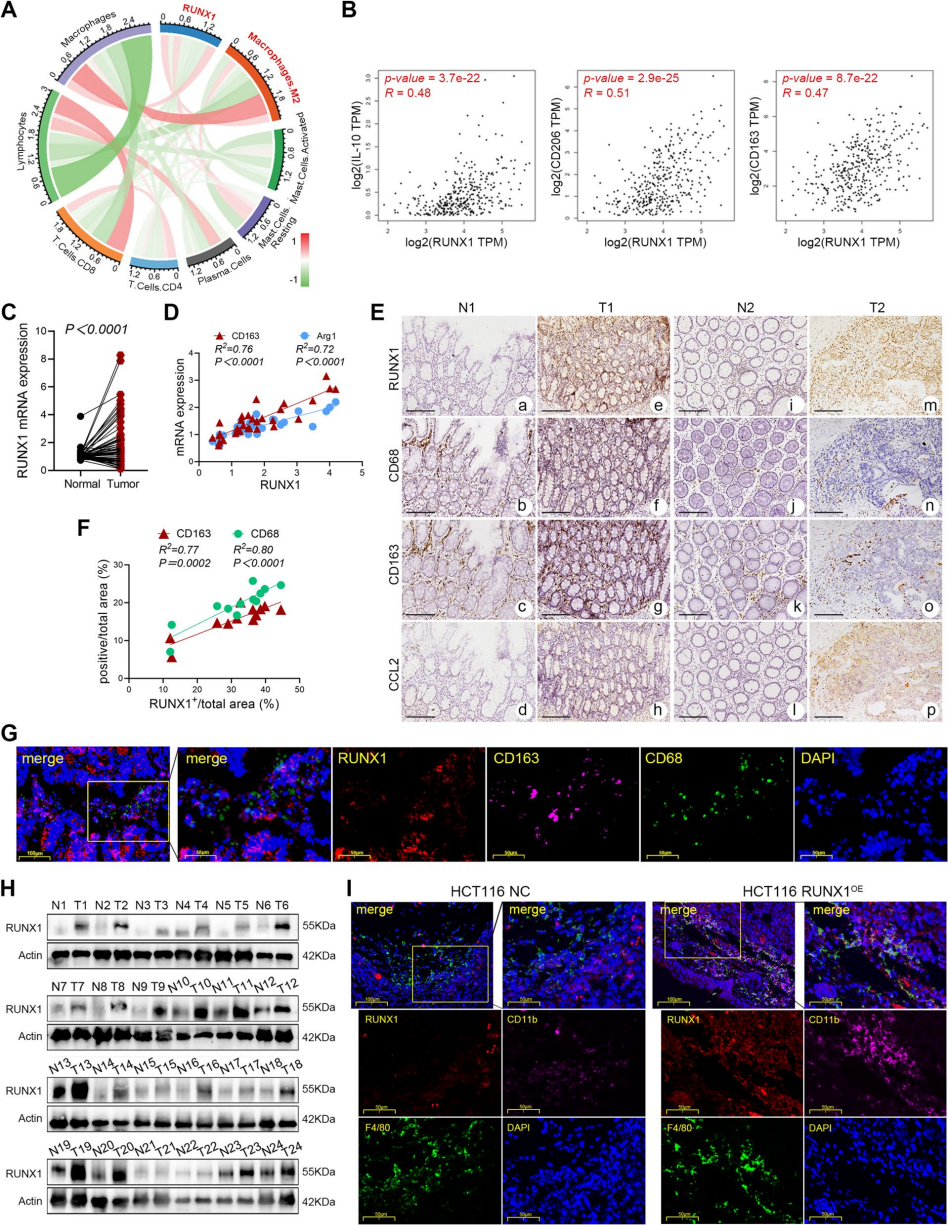
RUNX1 promotes TAMs infiltration in CRC. **A** Circular plot of immune cells enriched for the RUNX1-associated genes in COAD. RUNX1 expression is well related to M2 macrophage in COAD. **B** Correlation analysis between RUNX1 and IL-10 (left), CD206 (middle) and CD163 (right) in the TCGA database. **C** RT-qPCR analysis of the RUNX1 mRNA expression in tumor specimens and matched normal tissues (*n* = 66). **D** Correlation between the mRNA expression of RUNX1 and CD163, Arg1 in CRC tissues (*n* = 30). **E** Representative IHC analysis of RUNX1, CD68, CD163 and CCL2 expression in 12 pairs of cancer and the adjacent normal tissue from CRC patients. Scale bars: 100 μm. **F** Correlation between the expression of RUNX1 and CD68, CD163 in the IHC analysis. **G** Multi-label immunofluorescence staining of tumor tissue in patients with CRC. **H** The expression of RUNX1 in CRC tumor tissues and matched normal tissues was determined by western blotting (*n* = 24). **I** Multi-label immunofluorescence staining of tumor tissue from the nude mouse orthotopic CRC tumors

Bioinformatics analysis showed that RUNX1 was highly expressed in CRC, which was consistent with our previous study (Supplementary Fig. 1A-D) [12, 13]. The relationship between the expression of RUNX1 and various immune cell markers in CRC was analyzed by the public online website GEPIA (http://gepia.cancer-pku.cn.index.html). The results demonstrated that RUNX1 expression was significantly associated with a variety of immune cells, including macrophages (especially M2 macrophages) (Supplementary Table 2). In the disorder of COAD, the correlation was also explored on the TCGA (the cancer genome atlas) database via the CIBERSORT method, and the circular plot showed that the expression of RUNX1 was significantly positively correlated with the infiltration of macrophages (*P*<0.001,* R* = 0.26) and M2 macrophages (*P*<0.001, *R* = 0.24) in COAD (Fig. 1A). Further, RUNX1 was positively correlated with the expression of M2-TAMs related markers: CD206 (*P* = 2.9e-25, *R* = 0.51), CD163 (*P* = 8.7e-22, *R* = 0.47) and IL-10 (*P* = 3.7e-22, *R* = 0.48) (Fig. 1B). The tumor immune cell infiltration analysis, conducted on the online website TIMER (http://cistrome.shinyapps.io/timer/), suggested that the expression level of RUNX1 in CRC was positively correlated with the infiltration of TAMs (Supplementary Fig. 1E, Supplementary Table 3).

To explore the recruitment of TAMs in CRC tissue possibly related to RUNX1, we collected 66 pairs of fresh tumor specimens with matched normal tissues from CRC patients in our hospital. The qPCR analysis (*n* = 66) revealed an elevated expression of RUNX1 in tumor tissues (*P* < 0.0001, Fig. 1C), and the positive associations between RUNX1 mRNA and CD163 mRNA, Arg1 mRNA, CD68 mRNA, CD206 mRNA, IL-10 mRNA were observed (all *P* < 0.05, Fig. 1D, Supplementary Fig. 1F). Also, 20 out of 24 pairs of CRC samples presented with highly expressed RUNX1 in tumor tissue, as demonstrated by the western blotting analyses (Fig. 1H, Supplementary Fig. 1H). IHC analysis of 12 pairs of randomly selected CRC samples showed a positive correlation between the proportion of RUNX1 positive areas and CD68 (*P* < 0.0001), CD163 (*P* = 0.0002) positive areas in tumor (Fig. 1E-F). And the multi-label IF staining analysis of the tumor tissue from CRC patient suggested that the high expression of RUNX1 in tumor tissue is not caused by the abundance of macrophages (Fig. 1G). The RUNX1 associated accumulation of TAMs was further verified by the RUNX1-CD11b-F4/80 IF staining assay, which was conducted on the paraffin sections of the nude mouse orthotopic CRC tumors (Fig. 1I).

**Fig. 2:**
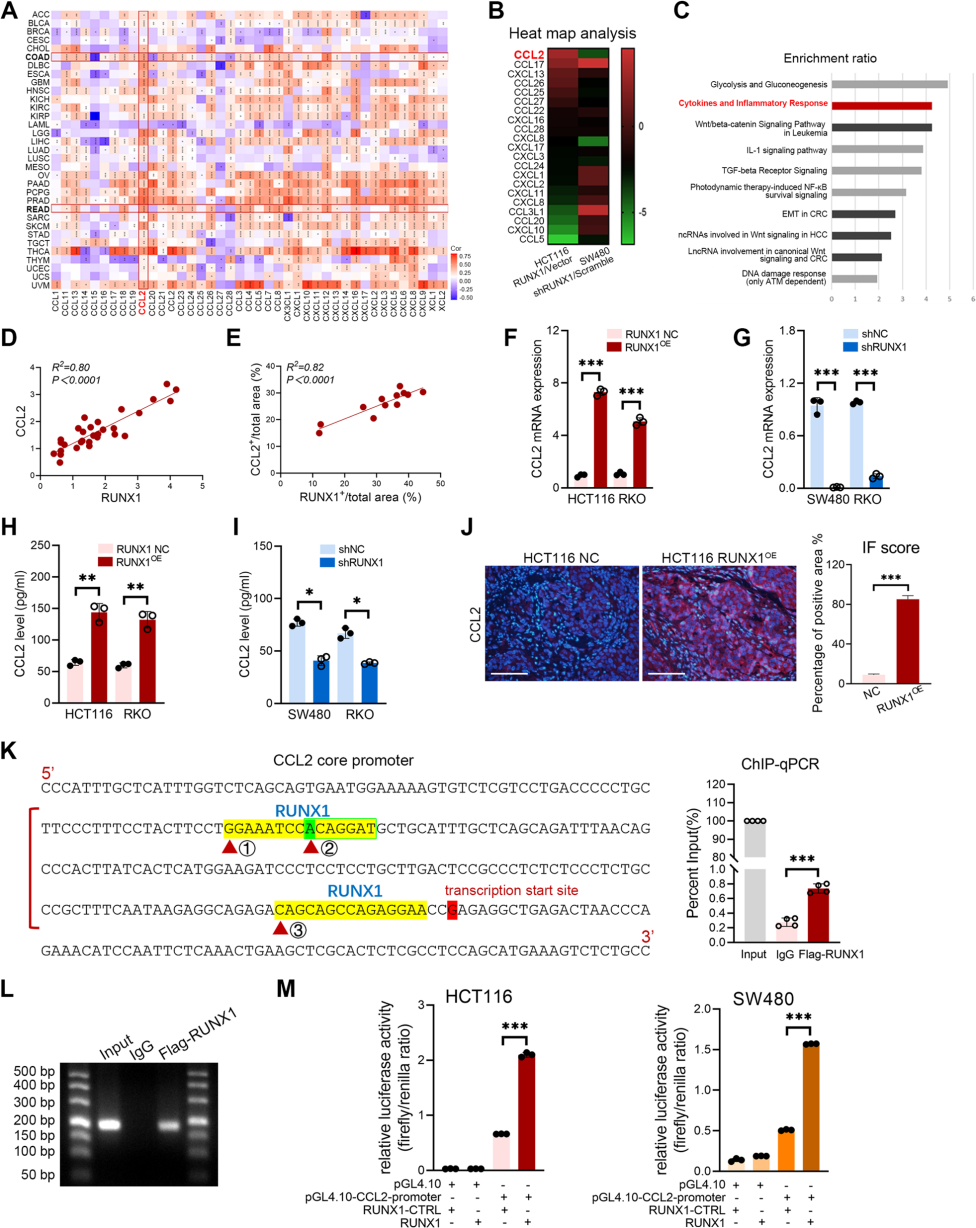
RUNX1 promotes CCL2-mediated recruitment of TAMs. **A** Correlation between the RUNX1 expression and chemokine family members in 33 TCGA tumors. **B** Heat map analysis of the chemokines and its receptors based on the RNA-Seq data of HCT116 RUNX1^OE^/vector and SW480 shRUNX1/scramble. **C** Enrichment analysis of RUNX1-related signaling pathways using Wikipathway Cancer platform. **D** Correlation analysis between the RUNX1 mRNA and CCL2 mRNA in tumor specimens (*n* = 30). **E** Correlation analysis between RUNX1 IHC staining and CCL2 staining in 12 pairs of CRC samples. **F**, **G** RT-qPCR analysis of the expression of CCL2 mRNA in stably transfected CRC cells. **H**, **I** ELISA analysis of the CCL2 generation in supernatant of RUNX1 over-expressed or knockdown CRC cells. **J** (left) Representative IF staining of CCL2 in the orthotopic CRC tumors with RUNX1 over-expressed. Scale bars: 200 μm. (right) Quantification of percentage of CCL2+ HCT116 cells (of HCT116 cells; *n* = 3). Quantitative data are indicated as mean ± SEM. **K** (left) Possible binding sites of RUNX1 in the CCL2 core promoter. (right) ChIP-qPCR assay in HCT116. **L** All PCR products were confirmed by DNA agarose gel electrophoresis. **M** Dual-Glo luciferase assay in HCT116 and SW480

Next, we explored the correlation between RUNX1 and chemokine family members in a total of 33 different tumors based on the data of TCGA. The heatmap data of the chemokine family members was supplied by the “Gene_Corr” module of Tumor Immune Estimation Resource 2 (TIMER2) web (http://timer.cistrome.org), which contained the partial Cor and *P*-value in the Spearman’s rank correlation test [29]. As shown in the heatmap of Fig. 2A, CCL2 expression was significantly correlated with RUNX1 both in COAD and READ (all *P* < 0.0001). Results of the heat map showed that CCL2 was positively associated with elevated RUNX1 in HCT116 RUNX1^OE^ cells, while it was negatively associated with decreased RUNX1 in SW480 shRUNX1 cells (Fig. 2B). RNA-Seq analysis also suggested that RUNX1 was closely correlated with the pathway related to “cytokines and inflammatory response” (Fig. 2C). And our findings further confirmed that RUNX1 is positively correlated with CCL2 both in the levels of transcription and translation in CRC tissues (all *P* < 0.0001, Fig. 1F s-x and Fig. 2D, E). ELISA and q-PCR analyses using stably transfected CRC cells showed that up-regulated RUNX1 promotes CCL2 expression, while down-regulated RUNX1 decreases (all *P* < 0.05, Fig. 2F-I and Supplementary Fig. 1G, I-L). *In vivo* work showed that the expression of CCL2 exhibits a positive correlation with that of RUNX1 in the orthotopic mouse model (*P* < 0.001, Fig. 2J). Then, we predicted the binding sites of RUNX1 in the CCL2 promoter region through online website EPD (https://epd.epfl.ch//index.php) and LASAGNA Search (https://biogrid-lasagna.engr.uconn.edu/lasagna_search/). And the results indicated that there may be three binding sites in the -160bp to 0bp interval of the CCL2 promoter fragment, as shown in Fig. 2K. Therefore, the ChIP-qPCR, DNA agarose gel electrophoresis, and dual-Glo luciferase assays were sequentially employed to explore the specific binding sites of RUNX1 in the CCL2 gene sequence (Fig. 2K-M). Our findings verified that RUNX1 can bind to the ACAGGAT region of CCL2 promoter, which is the second region in Fig. 2K. In a HCT116 cells-THP-1 co-culture system, we observed that macrophages in the HCT116 RUNX1^OE^/THP-1 co-culture group exhibted higher migration rate than the control group, and anti-CCL2 antibody (10 μg/ml) significantly attenuated the migration of M2 macrophages (all *P* < 0.05, Supplementary Fig. 2G).

### RUNX1 regulates M2 polarization of TAMs

**Fig. 3:**
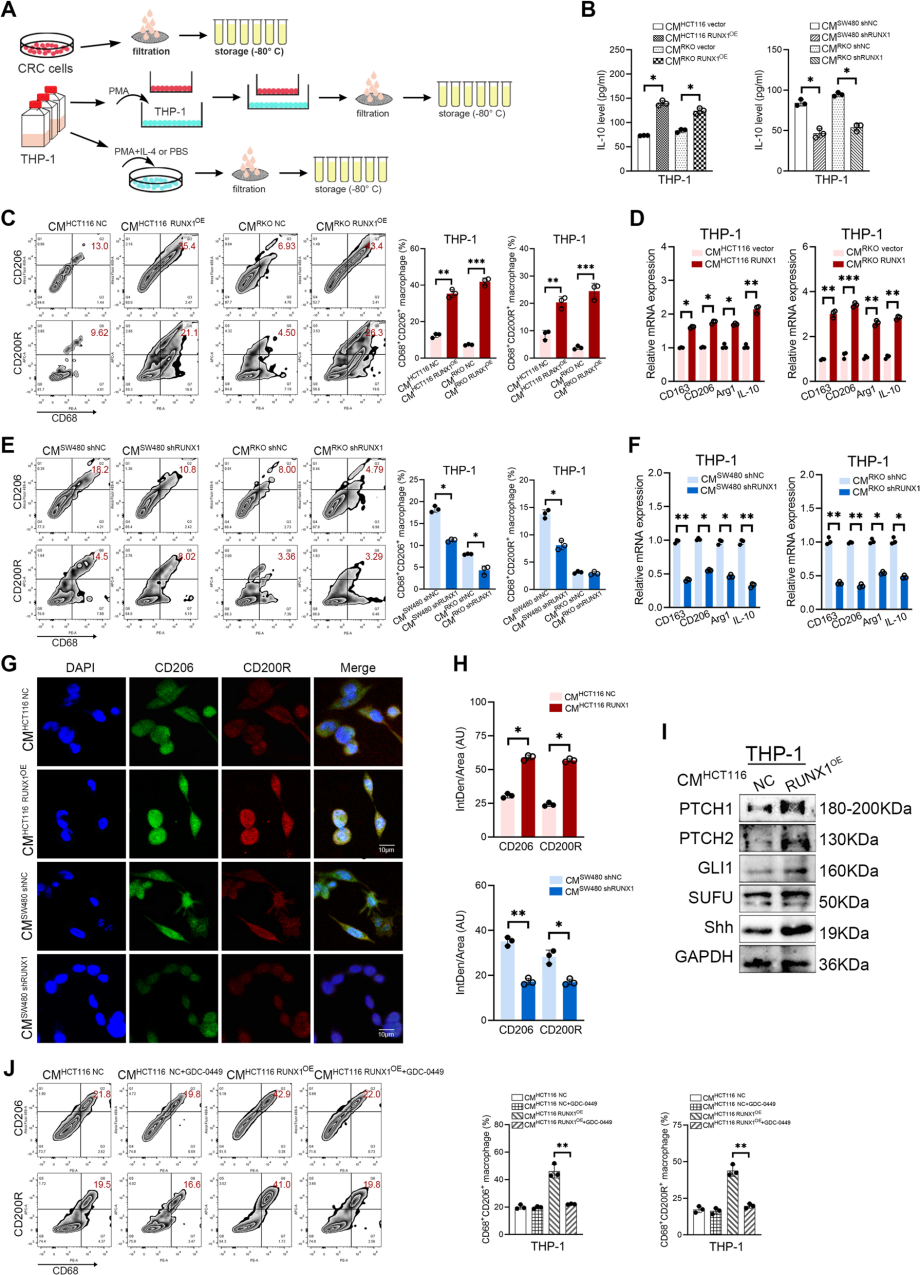
RUNX1 regulates M2 polarization of TAMs. **A** Experimental scheme of different CMs preparation and *in vitro* model of cells co-culture. **B** THP-1 was stimulated with CMs derived from RUNX1 over-expressed (left; HCT116 vector/RUNX1^OE^, RKO vector/RUNX1^OE^) or knockdown (right; SW480 shNC/shRUNX1, RKO shNC/shRUNX1) CRC cells, and the IL-10 in supernatant was analyzed by ELISA. **C** (left) Flow cytometry analysis of macrophage polarization phenotype stimulated by CMs derived from RUNX1 over-expressed HCT116 and RKO cells. (right) Quantification of percentage of CD68^+^CD206^+^ or CD68^+^CD200R^+^ macrophages (of macrophages; *n* = 3). **D** RT-qPCR analysis of the expression of CD163 mRNA, CD206 mRNA, Arg1 mRNA and IL-10 mRNA in macrophages treated with CMs derived from RUNX1 over-expressed HCT116 (left) and RKO (right) cells. (**E**) (left) Flow cytometry analysis of macrophage polarization state stimulated by CMs derived from RUNX1 knockdown SW480 and RKO cells. (right) Quantification of percentage of CD68^+^CD206^+^ or CD68^+^CD200R^+^ macrophages (of macrophages; *n* = 3). (**F**) RT-qPCR analysis of the expression of CD163 mRNA, CD206 mRNA, Arg1 mRNA and IL-10 mRNA in macrophages treated with CMs derived from RUNX1 knockdown SW480 (left) and RKO (right) cells. **G** CD206 and CD200R IF labeling of THP-1 cells treated with CRC cells-derived CMs. Data were recorded by a confocal laser scanning microscopy. Scale bars: 10 μm. **H** Quantification of mean fluorescence intensity of CD206 and CD200R (IntDen/Area; *n* = 3). **I** The expression of PTCH1, PTCH2, GLI1, SUFU and Shh in TAMs treated with CMs derived from RUNX1 over-expressed HCT116 cells was determined by western blotting. **J** (left) Flow cytometry analysis of macrophage phenotype induced by CMs derived from RUNX1 over-expressed HCT116 cells in the presence or absence of GDC-0449. (right) Quantification of percentage of CD68^+^CD206^+^ or CD68^+^CD200R^+^ macrophages (of macrophages; *n* = 3)

CMs derived from CRC cell lines were prepared as aforementioned (Fig. 3A). The IL-10 expression of THP-1 pre-stimulated by different CMs for 24 h was detected by ELISA. Data showed that macrophages treated with RUNX1 over-expression cells-derived CMs (HCT116 or RKO) secreted higher levels of IL-10 than the controls, while those treated with RUNX1 knockdown cells-derived CMs (SW480 or RKO) exhibited lower levels of IL-10 (all *P* < 0.05, Fig. 3B).

Flow cytometry was performed to monitor polarization phenotypes of THP-1. We noted that CMs from RUNX1 over-expressed CRC cells (HCT116 or RKO) significantly induce M2 polarization of THP-1 compared with control groups, which showed an increase in the proportion of CD68^+^CD206^+^ macrophage and CD68^+^CD200R^+^ macrophage (all *P* < 0.01, Fig. 3C). Intriguingly, the proportion of M2 polarized macrophages decreased when CMs from RUNX1 knockdown cell lines of SW480 and RKO were applied (Fig. 3E). Importantly, the polarization state of macrophages stimulated by RUNX1 over-expressing tumor cells was also examined in another cell model, primary bone marrow-derived macrophage (BMDMs). And our results showed that CT26-derived RUNX1 promoted an increase in CD68^+^CD206^+^ and CD68^+^CD200R^+^ double positive macrophages levels, consistent with the results in Figure 3C (all *P* < 0.05, Supplementary Fig. 2A-B). Moreover, the exogenous anti-CCL2 antibody significantly reduced the levels of RUNX1-dependent M2-BMDMs, indicating that chemokine CCL2 may play a key role in the polarization of M2-BMDMs *in vitro*. In a HCT116-THP-1 co-culture model, numbers of CD68^+^CD206^+^CD200R^+^ triple positive macrophages in the HCT116 RUNX1^OE^/THP-1 co-culture group significantly increased compared to the control group, indicating that RUNX1 overexpression in CRC cells skew the macrophage polarization toward M2 macrophage (*P* < 0.01, Supplementary Fig. 2F). The mRNA expression levels of CD163, CD206, Arg1 and IL-10 of THP-1 in different groups were analyzed by RT-qPCR, and findings were consistent with that of Flow cytometry (all *P* < 0.05, Fig. 3D, F). We conducted cyto IF double-staining to identify M2-TAM polarization phenotype, and results revealed that THP-1 cells treated with HCT116 RUNX1^OE^ cells-derived CMs expressed higher CD206 and CD200R than the control group, as shown in Fig. 3G, H. Similarly, THP-1 cells treated with SW480 shRUNX1 cells-derived CMs expressed lower CD206 and CD200R than the control group (Fig. 3G, H). Taken together, these findings support that CRC-derived RUNX1 promotes M2-TAM polarization *in vitro*. Western blotting assay showed that when THP-1 was stimulated with CMs derived from HCT116 RUNX1^OE^ cells, the expression of Hedgehog pathway-related molecules (PTCH1, PTCH2, GLI1, SUFU and Shh) were significantly higher than that in the control group, suggesting that the Hedgehog pathway of TAMs was activated in the current situation (Fig. 3I, Supplementary Fig. 2C). GDC-0449 (Vismodegib), a hedgehog pathway inhibitor (HPI), significantly reduces the proportion of double positive M2 macrophages *in vitro* (all *P* < 0.01, Fig. 3J).

RUNX1 induces crosstalk between CRC cells and TAMs to promote tumor angiogenesis

**Fig. 4:**
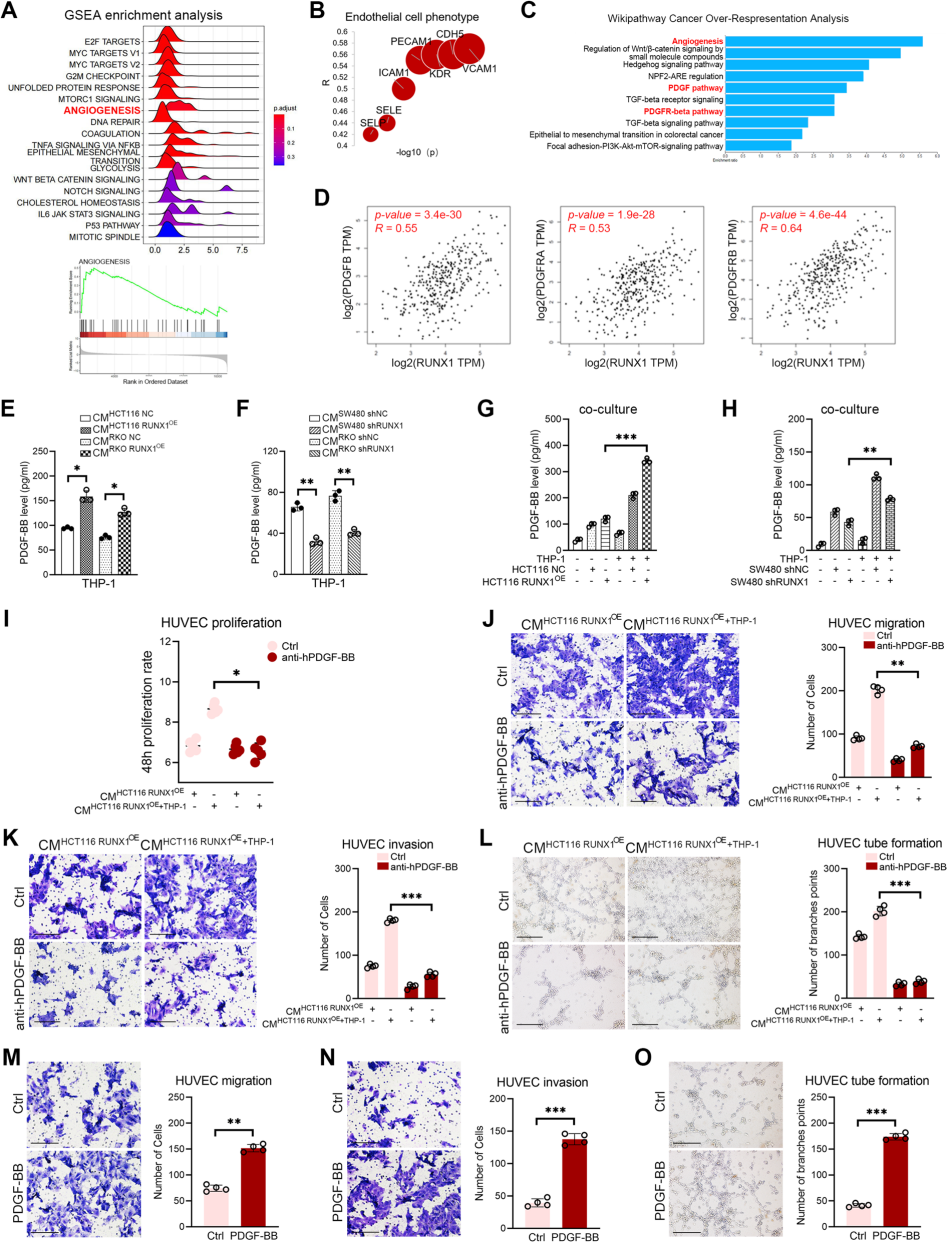
M2 TAMs derived PDGF-BB promotes tumor angiogenesis *in vitro*. **A** Gene set enrichment analysis in COAD conducted on the TCGA database. **B** Correlation analysis between RUNX1 and endothelial cell markers in CRC tissue samples. **C** Correlation analysis between RUNX1 and PDGFB (left), PDGFRA (middle) and PDGFRB (right) in the TCGA database. **D** Wikipathway cancer over-respresentation analysis of RUNX1 associated pathways. **E** The expression of PDGF-BB in THP-1 treated with CMs derived from RUNX1 over-expressed HCT116 and RKO cells was determined by ELISA. **F** The expression of PDGF-BB in THP-1 treated with CMs derived from RUNX1 down-regulated SW480 and RKO cells was determined by ELISA. **G** The expression of PDGF-BB in HCT116 RUNX1^OE^/THP-1 co-culture supernatant was determined by ELISA. **H** The expression of PDGF-BB in SW480 shRUNX1/THP-1 co-culture supernatant was determined by ELISA. **I** The 48 h proliferation rate of HUVECs treated with HCT116 RUNX1^OE^/THP-1 co-culture supernatant with or without anti-hPDGF-BB antibody (20 ng/ml) was detected. **J** (left) Transwell migration assay. The ability of HCT116 RUNX1^OE^/THP-1 co-culture supernatant to promote HUVECs migration in the presence or absence of anti-hPDGF-BB antibody (20 ng/ml) was evaluated. Scale bars: 200 μm. (right) Quantification of the number of cells migrating to the lower chamber. **K** (left) Transwell invasion assay. The ability of HCT116 RUNX1^OE^/THP-1 co-culture supernatant to promote HUVECs invasion in the presence or absence of anti-hPDGF-BB antibody (20 ng/ml) was evaluated. Scale bars: 200 μm. (right) Quantification of the number of cells invading to the lower chamber. **L** (left) Tube formation on Matrigel. The ability of HCT116 RUNX1^OE^/THP-1 co-culture supernatant to promote HUVECs tube formation in the presence or absence of anti-hPDGF-BB antibody (20 ng/ml) was evaluated. Scale bars: 200 μm. (right) Quantification of the branch points of HUVECs. **M** (left) Transwell migration assay. The ability of PDGF-BB to promote HUVECs migration was evaluated. Scale bars: 200 μm. (right) Quantification of the number of cells migrating to the lower chamber. **N** (left) Transwell invasion assay. The ability of PDGF-BB to promote HUVECs invasion was evaluated. Scale bars: 200 μm. (right) Quantification of number of cells invading to the lower well. **O** (left) Tube formation on Matrigel. The ability of PDGF-BB to promote HUVECs tube formation was examined. Scale bars: 200 μm. (right) Quantification of the branch points of HUVECs

To investigate the association between RUNX1 and angiogenesis, the top 300 RUNX1 expression-related genes was extracted from the TCGA data using the gene expression profiling interactive analysis 2 (GEPIA2) web server (http://gepia2.cancer-pku.cn/#analysis). And then the gene set enrichment analysis (GSEA) concerning these genes was conducted using an public online website WEB-based GEne SeT AnaLysis Toolkit (https://www.webgestalt.org/). Our results revealed that RUNX1 is associated with “angiogenesis” in CRC (Fig. 4A). Then, we explored the role of RUNX1 in CRC angiogenesis and analyzed its association with the expression of endothelial markers such as SELE, SELP, ICAM1, PECAM1, CDH5, KDR and VCAM1. The results suggested that RUNX1 was moderately correlated with the expression of these specific molecules in CRC (*R* ≥ 0.4, Fig. 4B). We further analysed the correlation between TAMs infiltration and angiogenesis in different stages of CRC tissues. And we found that the expression of CD31 was significantly positively correlated with that of CD68 and CD163 in stage II of CRC tissues (all *P* < 0.05, Supplementary Fig. 2D-E). The first 200 molecules with the strongest correlation with RUNX1 expression in CRC were further selected for Wikipathway cancer over-representation analysis via the WeGeatalt website (http://www.webgertalt.org/). The results showed that multiple pathways related to the malignant progression of CRC were enriched, including “angiogenesis”, “PDGF pathway” and “PDGFR-beta pathway” (Fig. 4C). Based on the correlation analysis of CRC tissue samples in TCGA database, it was found that RUNX1 was significantly correlated with PDGFB (*P* = 3.4e-30, *R* = 0.55), PDGFRA (*P* = 1.9e-28, *R* = 0.53) and PDGFRB (*P* = 4.6e-44, *R* = 0.64) (Fig. 4D). The level of PDGF-BB in M2 macrophage supernatant was detected by ELISA. The data showed that the concentration of PDGF-BB was positively correlated with the M2 polarization state of TAMs (all *P* < 0.05, Fig. 4E, F). It is worth noting that this observation was verified in the CRC cells-THP-1 co-culture model *in vitro* (all *P* < 0.01, Fig. 4G, H).

In order to further figure out the effect of CRC associated M2 macrophages on tumor angiogenesis, we cultured the HUVECs in the aforementioned HCT116 RUNX1^OE^/THP-1 co-culture-derived CMs with or without anti-hPDGF-BB antibody (20 ng/ml), and conducted the cell proliferation assay. Results suggested that M2 macrophages promoted the proliferation of HUVECs, and the proliferation rate of HUVECs treated with HCT116 RUNX1^OE^/THP-1 co-culture-derived CMs in the anti-hPDGF-BB group was significantly decreased than those in the control group (*P* < 0.05, Fig. 4I). Besides,* in vitro* studies such as the cell transwell migration and invasion assay and the tube formation on Matrigel assay showed that M2 macrophages regulated the migration (*P* < 0.01, Fig. 4J), invasion (*P* < 0.001, Fig. 4K) and tube formation (*P* < 0.001, Fig. 4L) of HUVECs. And the migration, invasion, and tube formation of HUVECs was markedly reduced in the presence of anti-hPDGF-BB antibody, indicating a strong link between endogenous PDGF-BB and vascular growth. Moreover, exogenous PDGF-BB also promoted endothelial cell migration (*P* < 0.01, Fig. 4M), invasion (*P* < 0.001, Fig. 4N) and tube formation (*P* < 0.001, Fig. 4O) *in vitro.* Taken together, these results indicated that M2-TAMs may promote tumor angiogenesis *in vitro* by secreting PDGF-BB.

**Fig. 5:**
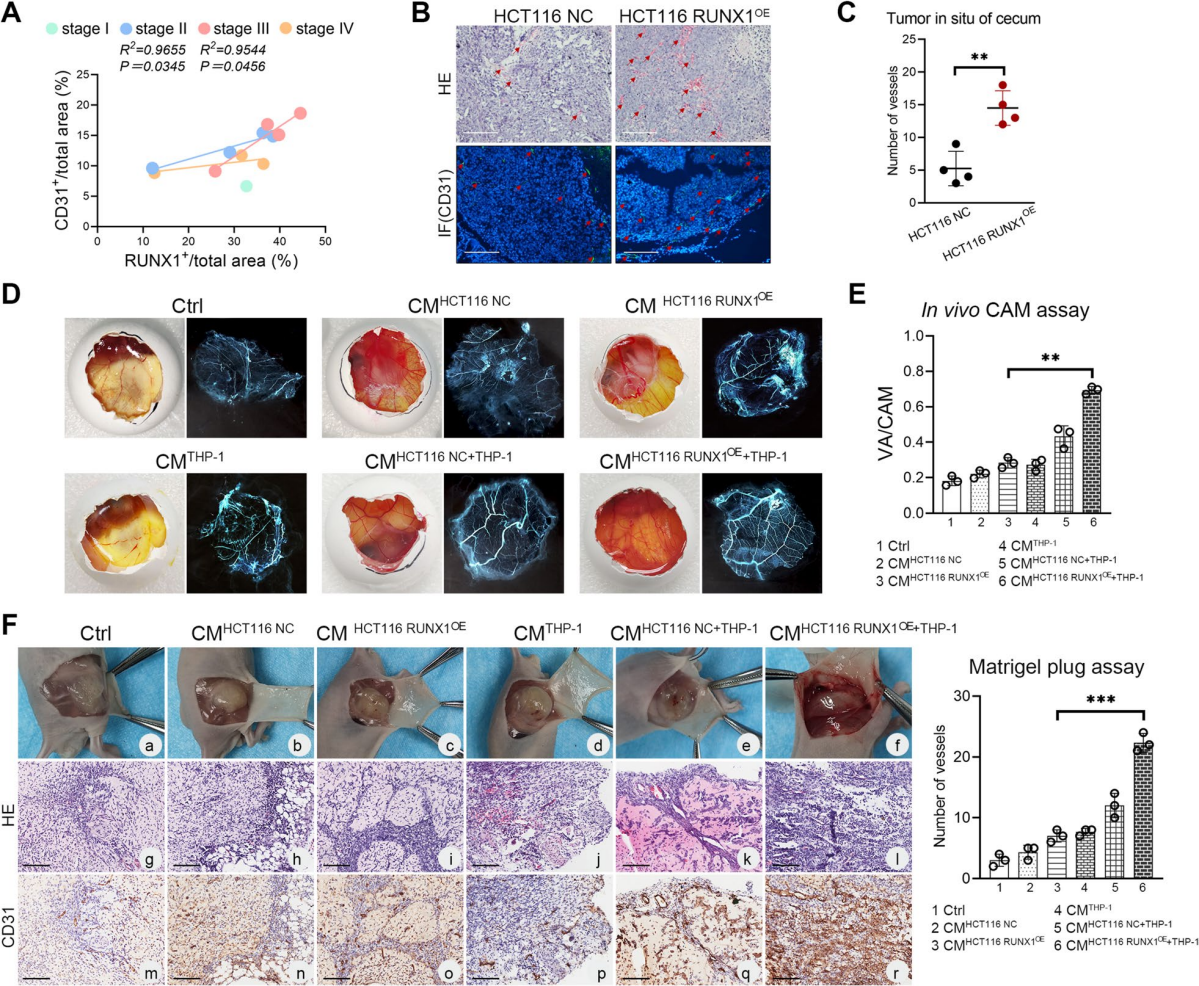
RUNX1 induces crosstalk between CRC cells and TAMs to promote tumor angiogenesis. **A** Correlation analysis of the expression of RUNX1 and CD31 in different stages of CRC tissues by IHC. **B** Representative H&E and CD31 IF analysis of orthotopic CRC tumors in nude mice. Scale bars: 200 μm. **C** The number of blood vessels in H&E sections of orthotopic tumor. (*n* = 4, *P* < 0.01). **D** *In vivo* CAM assay. Photographs of the CAM assay showing a 9^th^ day fertilized egg subjected to PBS, HCT116 NC-derived CMs, HCT116 RUNX1^OE^-derived CMs, THP-1-derived CMs, HCT116 NC/THP-1 co-culture-derived CMs, HCT116 RUNX1^OE^/THP-1 co-culture-derived CMs. **E** The ratio of vascular area to CAM area was calculated using the software ImageJ. **F** (left) Matrigel plug assay in nude mice. Representative H&E (g-l) and CD31 IHC (m-r) staining analysis of the viscous plugs. Scale bars: 100 μm. (right) The number of blood vessels in H&E sections of orthotopic tumor (*n* = 3, *P* < 0.001)

We performed IHC CD31 staining to explore whether angiogenesis correlated with the RUNX1 expression in CRC tissues. Our results showed positive correlations between the expression of CD31 and RUNX1 both in stage II and stage III of CRC tissues, suggesting that angiogenesis was positively correlated with the expression of RUNX1 in at least stage II and stage III of CRC tissues (all *P* < 0.05, Fig. 5A). The analysis of H&E and CD31 IF labeling of orthotopic CRC tumors in nude mice demonstrated that the number of blood vessels in HCT116 RUNX1^OE^ group was significantly higher than that in the control group (*P* < 0.01, Fig. 5B-C), which further confirmed our conclusion. *In vivo* CAM assay showed that compared with HCT116 RUNX1^OE^ cell derived CMs, THP-1 and HCT116 RUNX1^OE^ cell co-cultured CMs significantly stimulated chorioallantoic membrane angiogenesis, which manifested as a significantly enhanced ratio of vascular area to CAM area (*P* < 0.01, Fig. 5D-E). Consistent angiogenesis effect was also observed in the matrigel plug assay in nude mice, that is, the H&E and CD31 IHC staining analysis of the viscous plugs paraffin sections showed that a large number of blood vessels grew into the plugs in the co-cultured group of THP-1 and HCT116 RUNX1^OE^ cell, suggesting that M2 macrophage derived PDGF-BB significantly promoted vessel formation (*P* < 0.001, Fig. 5F).

M2-TAMs derived PDGF-BB promotes malignant biological behavior of CRC cells

**Fig. 6:**
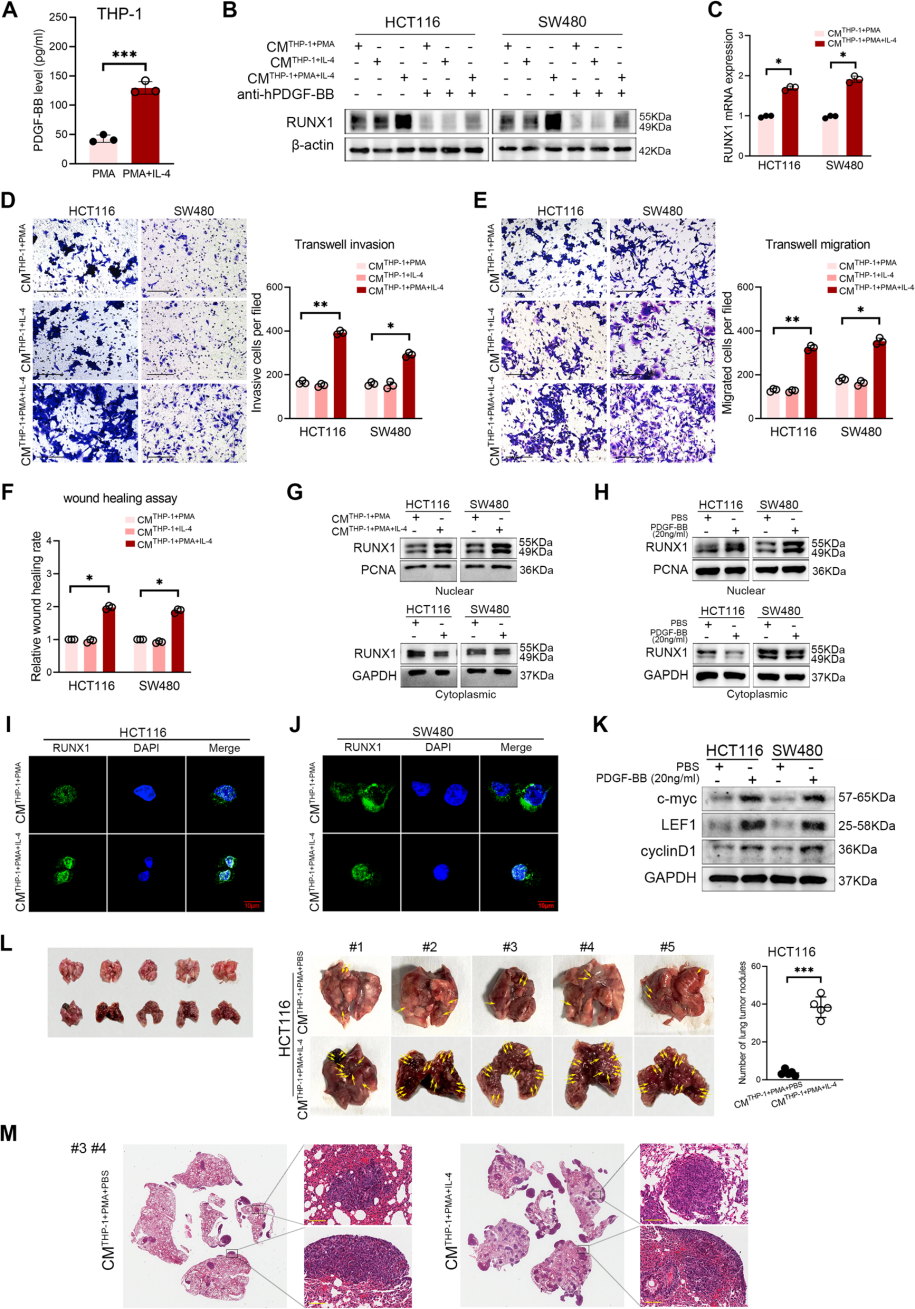
M2-TAMs derived PDGF-BB promotes malignant biological behavior of CRC cells **A** The levels of PDGF-BB in THP-1 culture medium with or without IL-4 stimulation were detected by ELISA. **B** The RUNX1 expression in HCT116 and SW480 cells treated with M0 or M2 TAMs culture supernatant in the presence or absence of anti-hPDGF-BB antibody were detected by western blotting. **C** RT-qPCR analysis of the RUNX1 mRNA expression in CRC cells treated with M0 or M2 TAMs culture supernatant. **D** (left) Transwell invasion assay. The invasion rate of HCT116 and SW480 cells treated with M0 or M2 TAMs culture supernatant were evaluated. Scale bars: 200 μm. (right) Quantification of the number of cells invading to the lower well. **E** (left) Transwell migration assay. The migration rate of HCT116 and SW480 cells treated with M0 or M2 TAMs culture supernatant were evaluated. Scale bars: 200 μm. (right) Quantification of the number of cells migrating to the lower chamber. **F** Wound healing assay. The ability of the M0 or M2 TAMs culture supernatant to promote CRC cells wound healing was evaluated. **G** The changes of RUNX1 expression in the cytosol and nuclei of HCT116 and SW480 cells upon the M0 or M2 TAMs culture supernatant stimulation were assessed by western blotting. **H** The changes of RUNX1 expression in the cytosol and nuclei of CRC cells treated with PBS or 20 ng/ml PDGF-BB were detected by western blotting. **I**, **J** RUNX1 IF labeling of HCT116 or SW480 cells treated with M0 or M2 TAMs culture supernatant. Data were recorded by confocal laser scanning. Scale bars: 10 μm. **K** The protein expression of c-myc, LEF1, cyclinD1 and GAPDH in HCT116 and SW480 cells treated with PBS or 20 ng/ml PDGF-BB were detected by western blotting. **L** The pulmonary metastasis model of CRC in nude mice. (left, middle) The full image of metastatic tumors. HCT116 cells were firstly stimulated with THP-1-derived CMs for 24h, and then inoculated into nude mice via caudal vein. (right) Quantification of numbers of the lung tumor nodules in different groups (*n* = 5). **M** Representative H&E staining analysis of the lung tumor nodules (#3, #4). Scale bars: 100 μm

Further, in order to clarify the potential effect of PDGF-BB on CRC cell lines, we detected the expression of RUNX1 in HCT116 and SW480 cells stimulated by THP-1 derived CMs for 24 h. The PMA differentiated-THP-1 macrophages (M0) were incubated with 100 ng/ml IL-4 *in vitro* to obtain M2 polarized macrophages. And ELISA analysis verified that macrophages in M2 polarization state secreted higher levels of PDGF-BB than M0 macrophages (*P* < 0.001, Fig. 6A). M2-BMDMs also exhibited higher levels of PDGF-BB than M0-BMDMs *in vitro* (*P* < 0.001, Supplementary Fig. 3A).

WB and qPCR analysis showed that M2 macrophages derived PDGF-BB enhanced the expression of RUNX1 in HCT116 and SW480 cells at the transcriptional and translational levels, respectively (Fig. 6B, C, Supplementary Fig. 2H). Consistent result was observed in the BMDMs cell model (Supplementary Fig. 3B-C). And the effect was reversed in the presence of 20 ng/ml anti-hPDGF-BB neutralizing antibody. It is of note that exogenous PDGF-BB can also promote the expression of RUNX1 in CRC cells in a concentration-dependent and time-dependent manner (Supplementary Fig. 2I-P). *In vitro* studies showed that PDGF-BB derived from M2 polarized macrophages modulated CRC cell invasion (all *P* < 0.05, Fig. 6D) and migration (all *P* < 0.05, Fig. 6E, F), while exogenous PDGF-BB also contributed to the malignant biological behaviors of CRC cells (all *P* < 0.05, Supplementary Fig. 3D-E). Moreover, in the BMDMs cell model, findings showed that M2-BMDMs-derived CMs modulated the migration and invasion of CT26 cells, and treatment of anti-hPDGF-BB neutralizing antibody (20 ng/ml) can reverse this trend (all *P* < 0.01, Supplementary Fig. 3F-G).

In this study, we investigated the effect of PDGF-BB on RUNX1 nuclear translocation in HCT116 and SW480 cells. The cells were treated with M0 or M2 macrophages derived CMs for 24 h, and RUNX1 protein expression was detected by western blotting. As shown in Fig. 6G and H, M2 macrophages derived or exogenous PDGF-BB markedly increased RUNX1 nuclear translocation (Supplementary Fig. 3H-I). Importantly, the RUNX1 nuclear aggregation of CRC cells induced by M2 macrophages derived PDGF-BB was observed by confocal laser scanning, as shown in Fig. 6I and J. With the treatment of 20 ng/ml PDGF-BB, the protein expression levels of c-myc, LEF1 and cyclinD1 in HCT116 and SW480 cells were significantly up-regulated, indicating the activation of Wnt/β-catenin signaling pathway (Fig. 6K, Supplementary Fig. 3J). To better flesh out our results, a pulmonary metastasis model of CRC in nude mice was successfully established with the HCT116 cells stimulated with THP-1-derived CMs. Our findings demonstrated that numbers of the lung tumor nodules in the CM^THP-1+PMA+IL-4^ group were significantly higher than those in the CM^THP-1+PMA+PBS^ group (*P* < 0.001, Fig. 6L). And H&E analysis of those metastatic nodules confirmed our findings (Fig. 6M), suggesting that M2 macrophages-derived CMs enhances the malignant biological behavior of CRC cells *in vivo*.

## Discussion

**Fig. 7:**
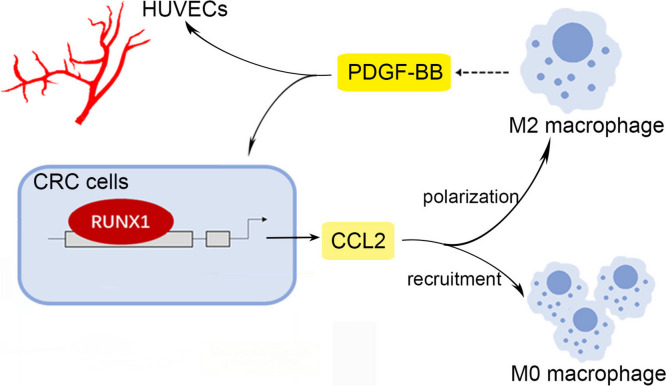
RUNX1 promotes angiogenesis in CRC by regulating the crosstalk between tumor cells and TAMs. Summary diagram showing a complex interaction in the crosstalk of CRC cells and TAMs. RUNX1 derived from CRC cells recruits macrophages and induces the M2 polarization phenotype. The latter contributes to tumor angiogenesis *in vivo* and *in vitro* and migration and invasion of CRC cells via PDGF-BB, so as to form a positive feedback loop to promote the progression of CRC

Our previous work illustrated that RUNX1 facilitates CRC proliferation, metastasis and chemotherapy resistance [12, 13]. We inadvertently found that the overexpression of RUNX1 was often accompanied by more neovascularization in the metastatic CRC model. Still, it is unknown whether RUNX1 increases M2 TAMs infiltration or contributes to angiogenesis in CRC. We thus aimed to investigate the RUNX1 mediated crosstalk between tumor cells and M2 TAMs in CRC, and its relationship with neoplastic angiogenesis. Here, we show that RUNX1 derived from CRC cells can recruit macrophages and induce a M2 polarized phenotype, which occurs via CCL2 secretion and Hedgehog signaling activation. Furthermore, we show by CMs stimulation and co-culture studies that M2 TAMs produced PDGF-BB promotes tumor angiogenesis and enhances the malignant biological behavior of CRC cells (Fig. 7). These findings suggest a putative combined TAMs-directed therapeutic approach to CRC, where one agent disrupts the secretion of PDGF-BB in M2 polarized macrophages by jamming the Hedgehog pathway and another agent selectively cripples the unsupported CRC cells.

TAMs are the major inflammatory component of the stroma of many solid tumors, and their important contributions to tumor growth and progression have been well documented [25, 30–32]. The differentiation of TAMs into M1 or M2 phenotype is regulated by various microenvironmental signals, including signals from tumor cells [26, 32]. M2 TAMs are alternatively activated macrophages, which are induced under the influence of IL-4, IL-10 or IL-13 [32]. CCL2 is one of the major factors required to facilitate and direct macrophage infiltration into target tissues [33]. This monocyte chemokine was reported to polarize macrophages toward a pro-tumoral, immunosuppressive phenotype in the TME [34, 35]. A recent study advocated that targeting tumor cell-derived CCL2 seems to be a feasible strategy to overcome bevacizumab resistance in Etv5^+^ CRC [36]. In the TME, M2 macrophages are strongly implicated in tumor progression, and their high-level infiltration is associated with poor prognosis of patients with CRC [16–19, 21, 25, 26, 37]. In the current study, we examined the expression of CCL2 and the recruitment of macrophages in CRC, and evaluated the specific polarization phenotype of TAMs (M1 and M2) in TME. Our results showed that RUNX1 derived from tumor cells recruits TAMs cells and induces macrophage polarization into the M2 state by promoting the secretion of CCL2 and the activation of Hedgehog signaling pathway. This observation is consistent with our previous discovery of the tumor-promoting role of RUNX1, reinforcing interest in RUNX1 as a therapeutic target in CRC.

TAMs have also been considered to support angiogenesis of malignancies [24, 30, 31]. Sierra et al. argued that TAMs-derived semaphorin 4D (Sema4D) promoted neoplastic angiogenesis and vessel maturation, suggesting a protumoral role of macrophages in the tumor stroma [31]. It was found that the signaling by the RAGE receptor in TAMs drives glioma angiogenesis in the TME [24]. The PDGF family generally includes four structurally related members, PDGF-AA, - BB, - CC and -DD. PDGF-BB is the only ligand that can activate both the tyrosine kinase receptors, PDGFR-α and -β [38, 39]. Genetic and biochemical studies have implicated that members of the PDGF family are important angiogenic factors [40–42]. In the disease of chronic lymphoblastic leukemia, PDGF indirectly regulates angiogenesis and disease progression by regulating vascular endothelial growth factor (VEGF) production in mesenchymal stromal cells [42]. M2 polarized TAMs express massive amounts of cytokines, including IL-6, IL-10, fibroblast growth factor (FGF1, FGF2), and angiogenic factors such as VEGF and PDGF [43, 44]. Therefore, we speculate whether TAMs derived PDGF-BB contributes to CRC angiogenesis. Here we have set up a CRC cells-TAMs co-culture model to assess whether PDGF-BB plays a direct role in promoting tumor angiogenesis and metastasis. Our data showed that the PDGF-BB secreted by M2 polarized TAMs promoted tumor angiogenesis *in vitro* and *in vivo*. Consequently, we infer that the vascular mechanism may partially mediate the progression and metastasis of CRC promoted by TAMs. Since vascular maturity and functionality are closely associated with tumor metastasis [45], further study with a focus on the TAMs mediated vascular mechanism would be of great importance and necessity.

In the TME, macrophages provide generous signals to tumor cells [46]. Due to the pivotal role of macrophages in tumor progression, it has aroused great interest in developing new therapeutic strategies for targeting TAMs. In the melanoma model resistant to anti-PD-1 checkpoint therapy, Etzerodt et al. found that the specific exhaustion of CD163^+^ TAMs mobilized inflammatory monocytes and promoted cytotoxic T cell-mediated tumor regression [28]. Strategies currently in development include the CCL2/CCR2 blockade, and targeting the macrophage colony-stimulating factor receptor, c-FMS, and CD115 [47–49]. PDGFs and PDGFRs (PDGFR-α, PDGFR-β) are overexpressed in various cancers including CRC [38, 50]. High PDGF-BB expression was detected in CRC patients compared to those with adenomas, while upregulated PDGFR α/β in the CRC stroma were involved in tumor growth, invasion, metastasis, and poor survival [38]. Epithelial tumors with high expression of PDGF-BB are more sensitive to specific PDGFRβ kinase inhibitors, suggesting an optimized selective treatment strategy [51]. However, the exact role of PDGF-BB in tumor cell response still remains to be solved. In the present study, we unexpectedly discovered that PDGF-BB stimulated the expression of RUNX1 in CRC cells and promoted its malignant phenotype of migration and invasion *in vitro*, thus forming a positive feedback loop of RUNX1 and PDGF-BB to promote tumor angiogenesis and metastasis. Despite the application of antiangiogenic drugs in recent years, treatment in patients with advanced CRC still faces several obstacles. Hence, we boldly speculate that a combined anti-RUNX1 and anti-PDGF-BB treatment may inhibit tumor angiogenesis and growth more effectively than single treatment in CRCs with high expression of RUNX1.

In conclusion, we have shown that chemokine CCL2 generation and Hedgehog pathway activation promote RUNX1-dependent macrophages recruitment and M2 polarization. Our data demonstrate a fundamental role for M2 polarized macrophages in promoting tumor angiogenesis. Our data further highlight the significant downstream effects of TAMs regulating CRC migration and invasion via PDGF-BB, arguing for a complex interaction in the crosstalk of CRC cells and TAMs. Accordingly, the use of PDGFs/PDGFRs antagonists in combination with the therapeutic strategy for targeting RUNX1 seems to be the practicable approach in the cancers therapy.

### Supplementary Information

**Additional file 1: Supplementary Fig. 1.** RUNX1 expression is upregulated in CRC. (A) Expression profiles of RUNX1 in diverse cancers and normal tissues using the Oncomine database (http://www.oncomine.org/resource/login.html). (B) Expression profiles of RUNX1 in diverse cancers and normal tissues using the online website TIMER (http://cistrome.shinyapps.io/timer/). (C) RUNX1 is upregulated in colorectal cancer using the GEPIA analysis (http://gepia.cancer-pku.cn/). (D) IHC data from the Human Protein Atlas Analysis (HPA) observed RUNX1 expression in normal colon and CRC tissues (http://www.Proteinatlas.org/). (E) Correlation between RUNX1 expression and macrophage infiltrations in COAD. (F) Correlation between the mRNA expression of RUNX1 and CD68, CD206, IL-10 in CRC tissues (*n* = 30). (G) The RUNX1 expression in 293T, FHC, SW480, SW620, HT29, HCT116, RKO and LoVo cells were detected by western blotting. (H) The full picture of Figure 1H. (I-L) Stably transfected CRC cells were generated. Western blotting and RT-qPCR analysis of the expression of RUNX1 in HCT116, RKO and SW480 cells.

**Additional file 2: Supplementary Fig. 2.** Exogenous PDGF-BB promotes RUNX1 mediated malignant biological behavior of CRC cells. (A) Flow cytometry analysis of macrophage polarization state stimulated by CT26 RUNX1^OE^-derived CMs with or without anti-CCL2 antibody. (B) Quantification of percentage of CD68^+^CD206^+^ or CD68^+^CD200R^+^ double positive macrophages (of macrophages; *n* = 3). (C) The full picture of Figure 3I. (D, E) Correlation analysis of the expression of CD31 and CD68, CD163 in different stages of CRC tissues by IHC. (F) (left) Flow cytometry analysis of macrophage polarization phenotype in a HCT116-THP-1 co-culture model. (right) Quantification of percentage of CD68^+^CD206^+^CD200R^+^ triple positive macrophages (of total macrophages; *n* = 3). (G) (left) Transwell migration assay. The ability of RUNX1 in HCT116 cells to promote macrophages migration in the presence or absence of anti-CCL2 antibody was evaluated. Scale bars: 200 μm. (right) Quantification of the number of macrophages migrating to the lower chamber. (H) The full picture of Figure 6B. (I-P) Exogenous PDGF-BB promotes the expression of RUNX1 in a concentration dependent and time-dependent manner. Western blotting and RT-qPCR analysis of the expression of RUNX1 in HCT116 (I-L) or SW480 (M-P) cells.

**Additional file 3: Supplementary Fig. 3.** Exogenous PDGF-BB promotes RUNX1 mediated malignant biological behavior of CRC cells. (A) The levels of PDGF-BB in BMDMs culture medium with or without IL-4 stimulation were detected by ELISA. (B) The expression of RUNX1 in CT26 cells treated with BMDMs-derived CMs was determined by western blotting. (C) RT-qPCR analysis of the RUNX1 mRNA expression in CT26 cells. Exogenous PDGF-BB contributes to the migration and invasion of HCT116 and SW480 cells* in vitro*, performed by the wound healing assay (D) and transwell invasion assay (E), respectively. Scale bars: 200 μm. (F) (left) Wound healing assay. (right) Quantification of the relative rate of wound healing of CT26 cells. (G) (left) Transwell invasion assay. Scale bars: 200 μm. (right) Quantification of the number of cells invading to the lower well. (H) The full picture of Figure 6G. (I) The full picture of Figure 6H. (J) The full picture of Figure 6K.

**Additional file 4.** 

**Additional file 5.** 

## Data Availability

Please contact author for data requests.

## References

[1] Sung H, Ferlay J, Siegel RL, Laversanne M, Soerjomataram I, Jemal A (2021). Global cancer statistics 2020: GLOBOCAN estimates of incidence and mortality worldwide for 36 cancers in 185 countries. CA Cancer J Clin.

[2] Teleanu RI, Chircov C, Grumezescu AM, Teleanu DM (2019). Tumor Angiogenesis and Anti-Angiogenic Strategies for Cancer Treatment. J Clin Med.

[3] Lugano R, Ramachandran M, Dimberg A (2020). Tumor angiogenesis: causes, consequences, challenges and opportunities. Cell Mol Life Sci.

[4] Folkman J (2007). Angiogenesis: an organizing principle for drug discovery?. Nat Rev Drug Discov.

[5] Kircher SM, Nimeiri HS, Benson AB (2016). Targeting Angiogenesis in Colorectal Cancer: Tyrosine Kinase Inhibitors. Cancer J.

[6] Hong D, Fritz AJ, Gordon JA, Tye CE, Boyd JR, Tracy KM (2019). RUNX1-dependent mechanisms in biological control and dysregulation in cancer. J Cell Physiol.

[7] Lie-A-Ling M, Mevel R, Patel R, Blyth K, Baena E, Kouskoff V (2020). RUNX1 Dosage in Development and Cancer. Mol Cells.

[8] Ito Y, Bae SC, Chuang LS (2015). The RUNX family: developmental regulators in cancer. Nat Rev Cancer.

[9] Rooney N, Mason SM, McDonald L, Däbritz JHM, Campbell KJ, Hedley A (2020). RUNX1 Is a Driver of Renal Cell Carcinoma Correlating with Clinical Outcome. Cancer Res.

[10] Deltcheva E, Nimmo R (2017). RUNX transcription factors at the interface of stem cells and cancer. Biochem J.

[11] Hong D, Fritz AJ, Finstad KH, Fitzgerald MP, Weinheimer A, Viens AL (2018). Suppression of Breast Cancer Stem Cells and Tumor Growth by the RUNX1 Transcription Factor. Mol Cancer Res.

[12] Li Q, Lai Q, He C, Fang Y, Yan Q, Zhang Y (2019). RUNX1 promotes tumour metastasis by activating the Wnt/β-catenin signalling pathway and EMT in colorectal cancer. J Exp Clin Cancer Res.

[13] Li Q, Lai Q, He C, Zhang H, Pan X, Li H (2021). RUNX1 regulates the proliferation and chemoresistance of colorectal cancer through the Hedgehog signaling pathway. J Cancer.

[14] Whiteside TL (2008). The tumor microenvironment and its role in promoting tumor growth. Oncogene.

[15] Mantovani A, Schioppa T, Porta C, Allavena P, Sica A (2006). Role of tumor-associated macrophages in tumor progression and invasion. Cancer Metastasis Rev.

[16] Hao NB, Lü MH, Fan YH, Cao YL, Zhang ZR, Yang SM (2012). Macrophages in tumor microenvironments and the progression of tumors. Clin Dev Immunol.

[17] Dijkgraaf EM, Heusinkveld M, Tummers B, Vogelpoel LT, Goedemans R, Jha V (2013). Chemotherapy alters monocyte differentiation to favor generation of cancer-supporting M2 macrophages in the tumor microenvironment. Cancer Res.

[18] Coussens LM, Werb Z (2002). Inflammation and cancer. Nature.

[19] Lewis CE, Pollard JW (2006). Distinct role of macrophages in different tumor microenvironments. Cancer Res.

[20] Coffelt SB, Hughes R, Lewis CE (2009). Tumor-associated macrophages: effectors of angiogenesis and tumor progression. Biochim Biophys Acta.

[21] Zhu C, Mustafa D, Zheng PP, van der Weiden M, Sacchetti A, Brandt M (2017). Activation of CECR1 in M2-like TAMs promotes paracrine stimulation-mediated glial tumor progression. Neuro Oncol.

[22] Chen X, Zhang L, Zhang IY, Liang J, Wang H, Ouyang M (2014). RAGE expression in tumor-associated macrophages promotes angiogenesis in glioma. Cancer Res.

[23] Prosniak M, Harshyne LA, Andrews DW, Kenyon LC, Bedelbaeva K, Apanasovich TV (2013). Glioma grade is associated with the accumulation and activity of cells bearing M2 monocyte markers. Clin Cancer Res.

[24] Gajewski TF, Schreiber H, Fu YX (2013). Innate and adaptive immune cells in the tumor microenvironment. Nat Immunol.

[25] Kinouchi M, Miura K, Mizoi T, Ishida K, Fujibuchi W, Sasaki H (2013). Infiltration of CD40-positive tumor-associated macrophages indicates a favorable prognosis in colorectal cancer patients. Hepatogastroenterology.

[26] Balkwill F, Mantovani A (2001). Inflammation and cancer: back to Virchow?. Lancet.

[27] Hu W, Li X, Zhang C, Yang Y, Jiang J, Wu C (2016). Tumor-associated macrophages in cancers. Clin Transl Oncol.

[28] Etzerodt A, Tsalkitzi K, Maniecki M, Damsky W, Delfini M, Baudoin E (2019). Specific targeting of CD163^+^ TAMs mobilizes inflammatory monocytes and promotes T cell-mediated tumor regression. J Exp Med.

[29] Li T, Fu J, Zeng Z, Cohen D, Li J, Chen Q (2020). TIMER2.0 for analysis of tumor-infiltrating immune cells. Nucleic Acids Res.

[30] Noy R, Pollard JW (2014). Tumor-associated macrophages: from mechanisms to therapy. Immunity.

[31] Sierra JR, Corso S, Caione L, Cepero V, Conrotto P, Cignetti A (2008). Tumor angiogenesis and progression are enhanced by Sema4D produced by tumor-associated macrophages. J Exp Med.

[32] Qian BZ, Pollard JW (2010). Macrophage diversity enhances tumor progression and metastasis. Cell.

[33] Cherry JD, Meng G, Daley S, Xia W, Svirsky S, Alvarez VE (2020). CCL2 is associated with microglia and macrophage recruitment in chronic traumatic encephalopathy. J Neuroinflammation.

[34] Sierra-Filardi E, Nieto C, Domínguez-Soto A, Barroso R, Sánchez-Mateos P, Puig-Kroger A (2014). CCL2 shapes macrophage polarization by GM-CSF and M-CSF: identification of CCL2/CCR2-dependent gene expression profile. J Immunol.

[35] Zheng Y, Wang Z, Wei S, Liu Z, Chen G (2021). Epigenetic silencing of chemokine CCL2 represses macrophage infiltration to potentiate tumor development in small cell lung cancer. Cancer Lett.

[36] Feng H, Liu K, Shen X, Liang J, Wang C, Qiu W (2020). Targeting tumor cell-derived CCL2 as a strategy to overcome Bevacizumab resistance in ETV5^+^ colorectal cancer. Cell Death Dis.

[37] Cheng Y, Zhu Y, Xu J, Yang M, Chen P, Xu W (2018). PKN2 in colon cancer cells inhibits M2 phenotype polarization of tumor-associated macrophages via regulating DUSP6-Erk1/2 pathway. Mol Cancer.

[38] Manzat Saplacan RM, Balacescu L, Gherman C, Chira RI, Craiu A, Mircea PA (2017). The Role of PDGFs and PDGFRs in Colorectal Cancer. Mediators Inflamm.

[39] Kazlauskas A (2017). PDGFs and their receptors. Gene.

[40] Chen L, Wu H, Ren C, Liu G, Zhang W, Liu W (2021). Inhibition of PDGF-BB reduces alkali-induced corneal neovascularization in mice. Mol Med Rep.

[41] Roskoski R (2018). The role of small molecule platelet-derived growth factor receptor (PDGFR) inhibitors in the treatment of neoplastic disorders. Pharmacol Res.

[42] Ding W, Knox TR, Tschumper RC, Wu W, Schwager SM, Boysen JC (2010). Platelet-derived growth factor (PDGF)-PDGF receptor interaction activates bone marrow-derived mesenchymal stromal cells derived from chronic lymphocytic leukemia: implications for an angiogenic switch. Blood.

[43] Xiao X, Gaffar I, Guo P, Wiersch J, Fischbach S, Peirish L (2014). M2 macrophages promote beta-cell proliferation by up-regulation of SMAD7. Proc Natl Acad Sci U S A.

[44] Chernykh ER, Shevela EY, Starostina NM, Morozov SA, Davydova MN, Menyaeva EV (2016). Safety and Therapeutic Potential of M2 Macrophages in Stroke Treatment. Cell Transplant.

[45] Carmeliet P, Jain RK (2011). Principles and mechanisms of vessel normalization for cancer and other angiogenic diseases. Nat Rev Drug Discov.

[46] Charles NA, Holland EC, Gilbertson R, Glass R, Kettenmann H (2012). The brain tumor microenvironment. Glia.

[47] Yang L, Zhang Y (2017). Tumor-associated macrophages, potential targets for cancer treatment. Biomark Res.

[48] Lim SY, Yuzhalin AE, Gordon-Weeks AN, Muschel RJ (2016). Targeting the CCL2-CCR2 signaling axis in cancer metastasis. Oncotarget.

[49] Li X, Yao W, Yuan Y, Chen P, Li B, Li J (2017). Targeting of tumour-infiltrating macrophages via CCL2/CCR2 signalling as a therapeutic strategy against hepatocellular carcinoma. Gut.

[50] Wang JC, Li GY, Wang B, Han SX, Sun X, Jiang YN (2019). Metformin inhibits metastatic breast cancer progression and improves chemosensitivity by inducing vessel normalization via PDGF-B downregulation. J Exp Clin Cancer Res.

[51] Tsioumpekou M, Cunha SI, Ma H, Åhgren A, Cedervall J, Olsson AK (2020). Specific targeting of PDGFRβ in the stroma inhibits growth and angiogenesis in tumors with high PDGF-BB expression. Theranostics.

