# Supplementary figures and images for: RUNX1 promotes angiogenesis in colorectal cancer by regulating the crosstalk between tumor cells and tumor associated macrophages
Source: Biomark Res. 2024 Feb 28;12:29. doi: 10.1186/s40364-024-00573-1 (PMC10903076; doi:10.1186/s40364-024-00573-1)

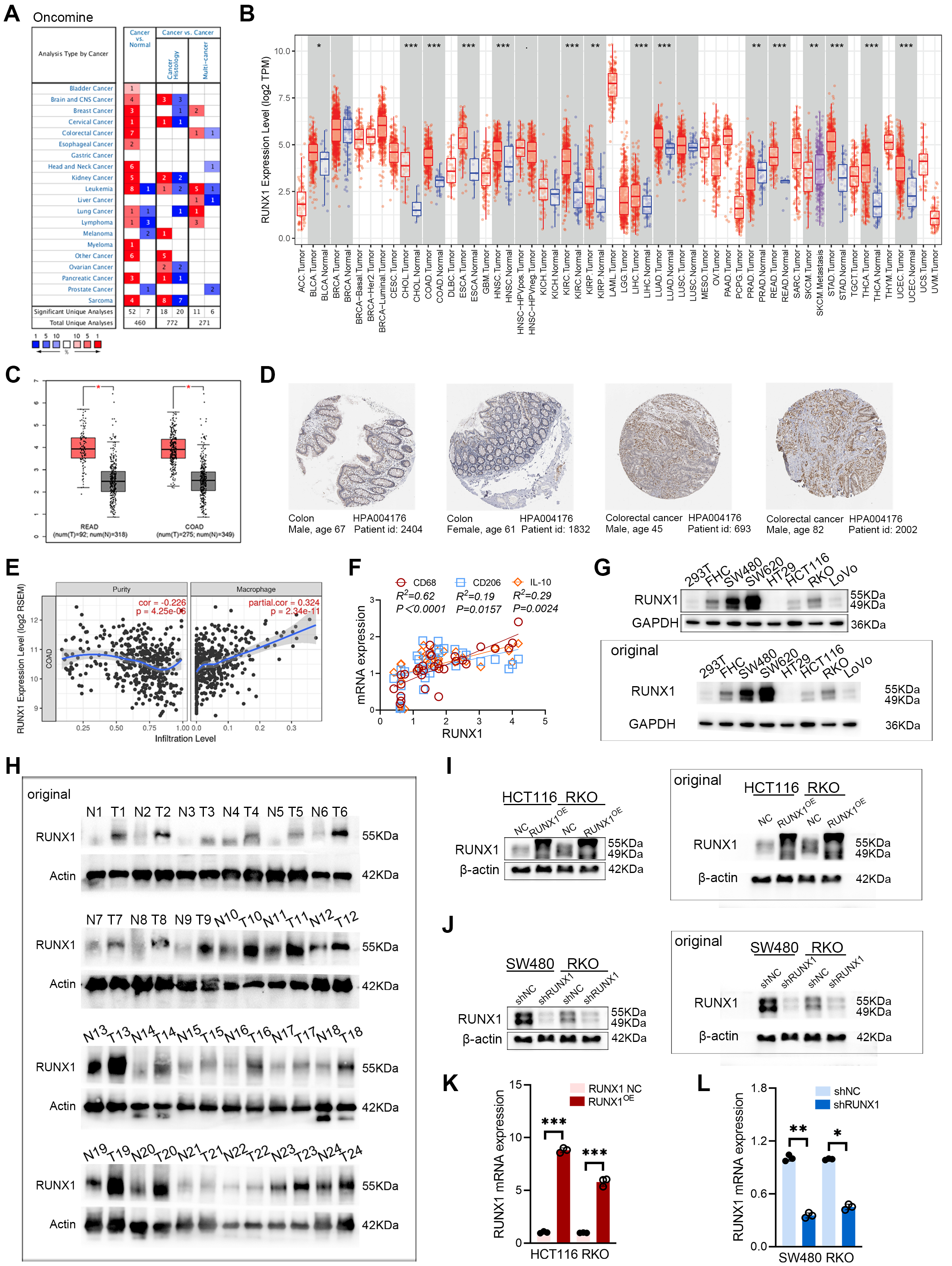

Supplement: Supplementary file 1 — Additional file 1: Supplementary Fig. 1. RUNX1 expression is upregulated in CRC. (A) Expression profiles of RUNX1 in diverse cancers and normal tissues using the Oncomine database (http://www.oncomine.org/resource/login.html). (B) Expression profiles of RUNX1 in diverse cancers and normal tissues using the online website TIMER (http://cistrome.shinyapps.io/timer/). (C) RUNX1 is upregulated in colorectal cancer using the GEPIA analysis (http://gepia.cancer-pku.cn/). (D) IHC data from the Human Protein Atlas Analysis (HPA) observed RUNX1 expression in normal colon and CRC tissues (http://www.Proteinatlas.org/). (E) Correlation between RUNX1 expression and macrophage infiltrations in COAD. (F) Correlation between the mRNA expression of RUNX1 and CD68, CD206, IL-10 in CRC tissues (n = 30). (G) The RUNX1 expression in 293T, FHC, SW480, SW620, HT29, HCT116, RKO and LoVo cells were detected by western blotting. (H) The full picture of Figure 1H. (I-L) Stably transfected CRC cells were generated. Western blotting and RT-qPCR analysis of the expression of RUNX1 in HCT116, RKO and SW480 cells. [file 40364_2024_573_MOESM1_ESM.tif]

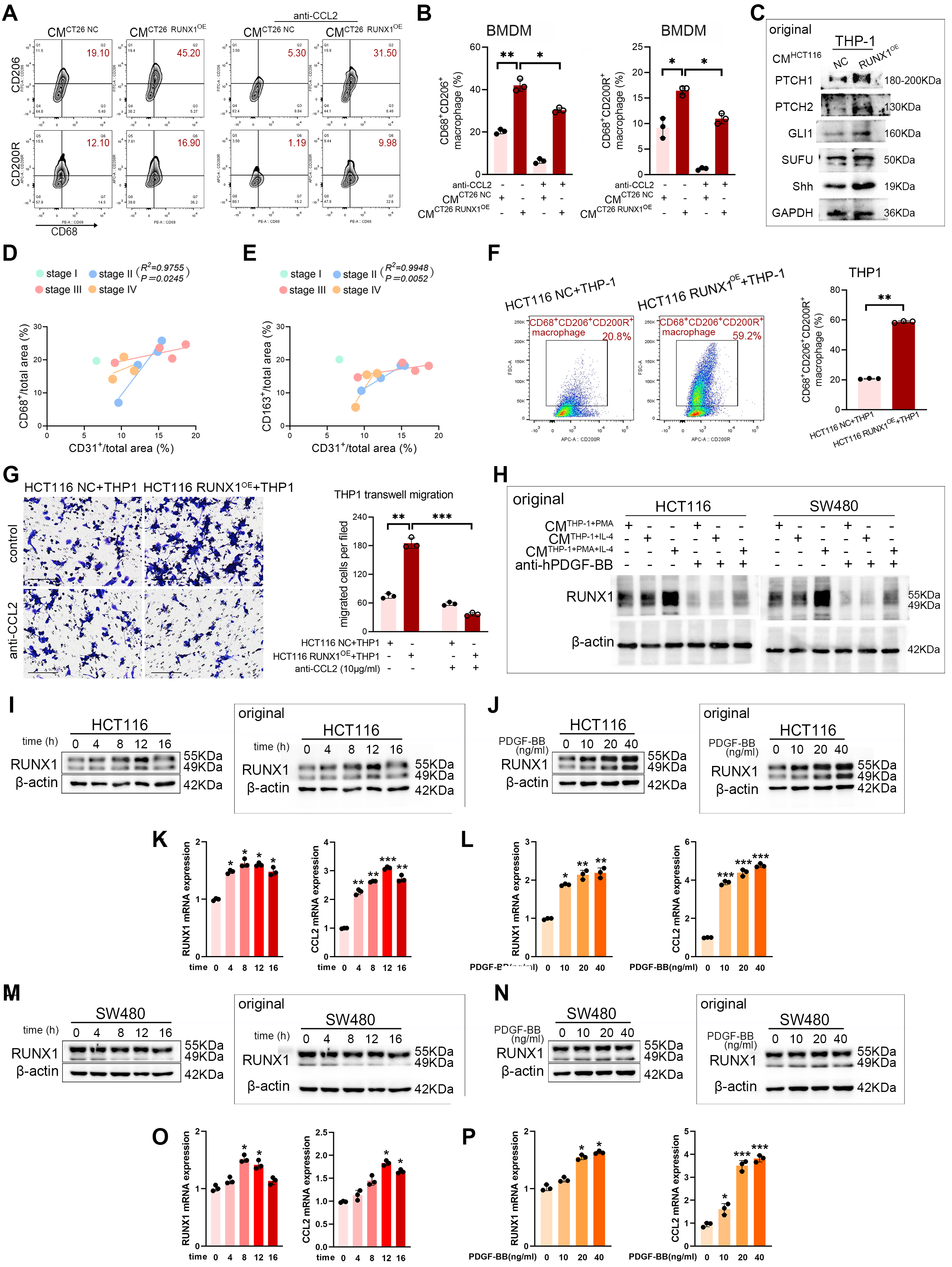

Supplement: Supplementary file 2 — Additional file 2: Supplementary Fig. 2. Exogenous PDGF-BB promotes RUNX1 mediated malignant biological behavior of CRC cells. (A) Flow cytometry analysis of macrophage polarization state stimulated by CT26 RUNX1OE-derived CMs with or without anti-CCL2 antibody. (B) Quantification of percentage of CD68+CD206+ or CD68+CD200R+ double positive macrophages (of macrophages; n = 3). (C) The full picture of Figure 3I. (D, E) Correlation analysis of the expression of CD31 and CD68, CD163 in different stages of CRC tissues by IHC. (F) (left) Flow cytometry analysis of macrophage polarization phenotype in a HCT116-THP-1 co-culture model. (right) Quantification of percentage of CD68+CD206+CD200R+ triple positive macrophages (of total macrophages; n = 3). (G) (left) Transwell migration assay. The ability of RUNX1 in HCT116 cells to promote macrophages migration in the presence or absence of anti-CCL2 antibody was evaluated. Scale bars: 200 μm. (right) Quantification of the number of macrophages migrating to the lower chamber. (H) The full picture of Figure 6B. (I-P) Exogenous PDGF-BB promotes the expression of RUNX1 in a concentration dependent and time-dependent manner. Western blotting and RT-qPCR analysis of the expression of RUNX1 in HCT116 (I-L) or SW480 (M-P) cells. [file 40364_2024_573_MOESM2_ESM.tif]

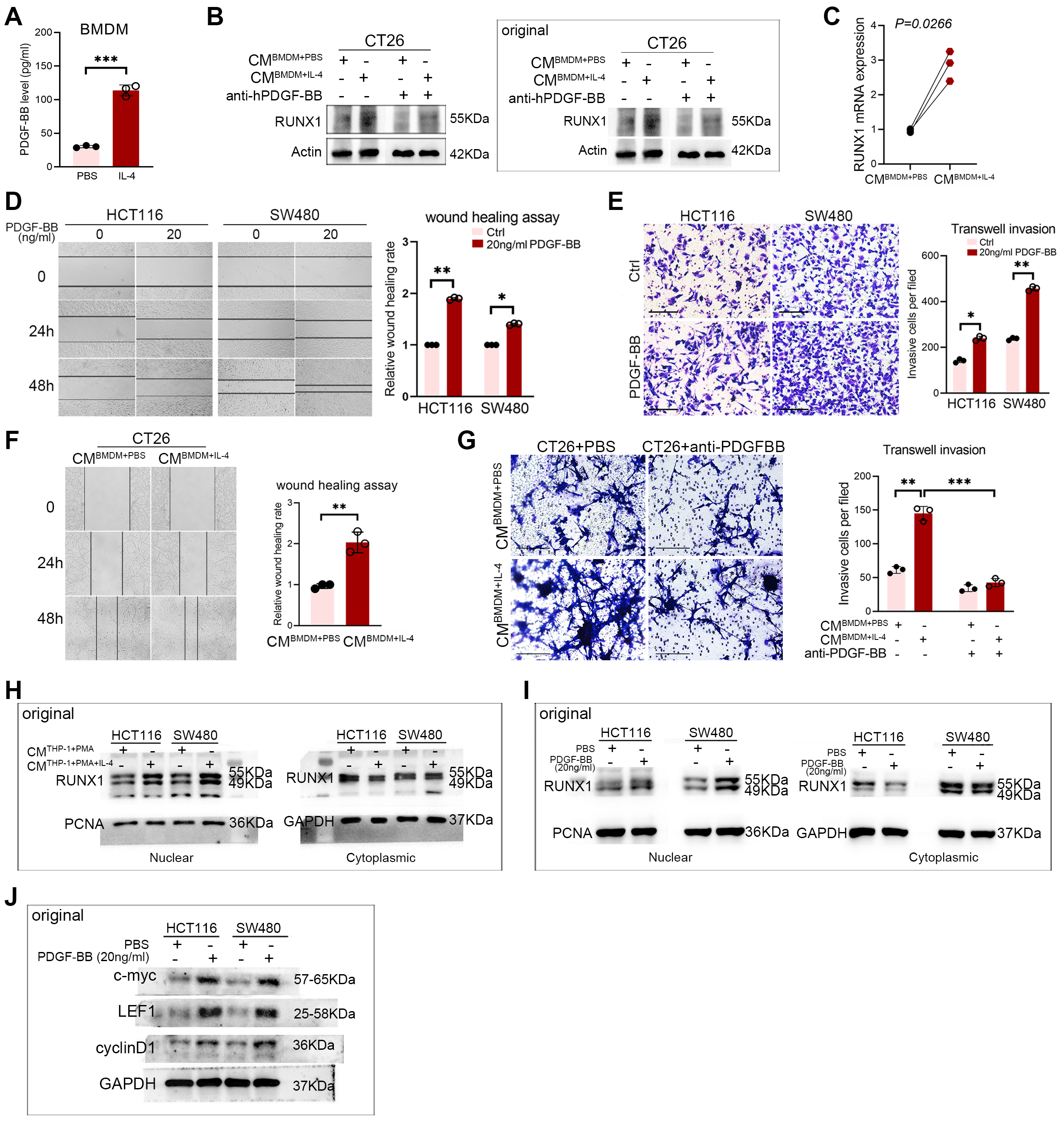

Supplement: Supplementary file 3 — Additional file 3: Supplementary Fig. 3. Exogenous PDGF-BB promotes RUNX1 mediated malignant biological behavior of CRC cells. (A) The levels of PDGF-BB in BMDMs culture medium with or without IL-4 stimulation were detected by ELISA. (B) The expression of RUNX1 in CT26 cells treated with BMDMs-derived CMs was determined by western blotting. (C) RT-qPCR analysis of the RUNX1 mRNA expression in CT26 cells. Exogenous PDGF-BB contributes to the migration and invasion of HCT116 and SW480 cells in vitro, performed by the wound healing assay (D) and transwell invasion assay (E), respectively. Scale bars: 200 μm. (F) (left) Wound healing assay. (right) Quantification of the relative rate of wound healing of CT26 cells. (G) (left) Transwell invasion assay. Scale bars: 200 μm. (right) Quantification of the number of cells invading to the lower well. (H) The full picture of Figure 6G. (I) The full picture of Figure 6H. (J) The full picture of Figure 6K. [file 40364_2024_573_MOESM3_ESM.tif]
