# Supplementary material for: RUNX1 promotes angiogenesis in colorectal cancer by regulating the crosstalk between tumor cells and tumor associated macrophages
Source: Biomark Res. 2024 Feb 28;12:29. doi: 10.1186/s40364-024-00573-1 (PMC10903076; doi:10.1186/s40364-024-00573-1)
Supplement: Supplementary file 4 — Additional file 4. [file 40364_2024_573_MOESM4_ESM.docx]

**Supplemental Methods**

**Cell culture and reagents**

HCT116 (RRID: CVCL_0291), SW480 (RRID: CVCL_0546), RKO (RRID: CVCL_0504) and THP-1 (RRID: CVCL_0006) cell lines were purchased from the Cell Bank of Type Culture Collection (CBTCC, Chinese Academy of Sciences, Shanghai, China). All cell lines were free of mycoplasma contamination (tested by the PCR). Cells were cultured in Dulbecco’s modified Eagle medium (DMEM) (Gibco, Carlsbad, CA) supplemented with 10% fetal bovine serum (FBS; Gibco, Carlsbad, CA) in a humidified 5% CO_2_ air atmosphere. Neutralizing antibody to hPDGF-BB (anti-hPDGF-BB, AF-220-NA) was purchased from R&D Systems. Recombinant Human PDGF-BB (100-14B) and Recombinant Human Interleukin-4 (IL-4) (200-04) were purchased from Pepro Tech. ELISA MAX™ Deluxe Set Human MCP-1/CCL2 Kit (438804) and ELISA MAX™ Deluxe Set Human Interleukin-10 (IL-10) Kit (430604) were purchased from BioLegend. Human PDGF-BB ELISA Kit (EK9137) was purchased from MultiSciences, and GDC-0449 (Vismodegib, S1082) from Selleck Chemicals, phorbol 12-myristate 13-acetate (PMA, HY-18739/CS-6053) from MedChemExpress, Nuclear-Cytosol Extraction Kit (FD0199) from Fudebio-tech.

**Stable cell lines generation**

Firstly, we detected the constitutive expression levels of RUNX1 protein in 8 different cell lines (Supplementary Fig. 1F). Then, we chose HCT116 and RKO cells for RUNX1 overexpression, and SW480 and RKO cells for RUNX1 knockdown. RUNX1 overexpression and knockdown were performed using a lentiviral packaging system. To construct overexpressing exogenous RUNX1 cell lines, full-length RUNX1 (NM_001754) was cloned into the expression vector pLenti-EF1a-EGFP-P2APuro-CMV (Obio Technology, Shanghai, China) and transfected into HCT116 and RKO cell lines according to the manufacturer’s instructions. Knockdown of endogenous RUNX1 was mediated by designed short hairpin RNAs (shRNAs) (Cyagen, Guangzhou, China) that were transfected into SW480 and RKO cell lines according to the manufacturer’s instructions. The RUNX1 shRNA sequence was sense 5′-CCAGGTTGCAAGATTTAAT-3′ and 5′-GGCAGAAACTAGATGATCA-3′, and the scramble sequence was sense 5′-CCTAAGGTTAAGTCGCCCTCG-3′. Stably transduced CRC cells were screened with 2 μg/ml puromycin (#EZ2811D376, BioFroxx, Germany) and finally maintained with 1 μg/ml puromycin.

**Bone marrow-derived macrophages (BMDMs)**

Primary BMDMs from Balb/c mice were cultured for 7 days in DMEM medium containing 10% FBS (Gibco, Carlsbad, CA), 30% L929 cell supernatant containing macrophage colony-stimulating factor (M-CSF), 200 mM glutamine (Sigma-Aldrich, Saint Louis, MO, United States) and 10 μg/mL ciprofloxacin (Isofarma, Eusébio, CE, BR). Mature, adherent BMDM were detached using 0.05% EDTA/PBS (Gibco, Waltham, MA, United States). *In vitro* experiments concerning BMDMs were thus performed. To prepare BMDMs-derived CMs, freshly isolated macrophages were cultured for 24 h in the presence of IL-4 (100 ng/ml) or PBS, then washed twice with PBS, and cultured in fresh DMEM complete medium for another 24 h. Finally, the culture medium was filtered and stored at -80 ℃ for follow-up usage.

**Cell proliferation assay**

HUVEC were seeded into a 96-well plate at 1,000 cells per well and then treated with the following CMs: HCT116 NC-derived supernatants, HCT116 RUNX1^OE^-derived supernatants, THP-1-derived supernatants, HCT116 NC-THP-1 co-culture-derived supernatants and HCT116 RUNX1^OE^-THP-1 co-culture-derived supernatants. For the cell proliferation assay, the cells were incubated with 10 μL of Cell Counting Kit-8 (CCK-8, Dojindo, Japan) for 2 h at 37°C and assessed by the Paradigm Detection Platform (Beckman, CA, USA). The cells were further incubated for 48 h following the CCK-8 assay. Each group was assessed in replicates of five wells.

**Cellular wound healing assay**

Cells were seeded on six-well culture plates at a density of approximately (1 × 10^6^) cells per well and incubated for 24 h (80 to 90% confluence). Scratch wounds were produced by a 10 μl plastic pipette tip, and then cells were cultured in THP-1-derived supernatants or in DMEM with 20 ng/ml PDGF-BB and 2% FBS. Wound margins were photographed, and migration was monitored after 48 h of wound formation. Cell motility was quantified by measuring the distance between the advancing margins of cells in three randomly selected microscopic fields (× 200) at each time point.

**Cell Transwell migration and invasion assays**

Approximately (5 × 10^4^) cells were suspended in 100 μl serum-free medium and seeded in the upper 8-μm polycarbonate nucleopore chambers (3422, Corning, USA), and the lower chambers were filled with 500 μl CMs with different treatments (unless otherwise stated). For the cell invasion assay, the Transwell chambers were Matrigel-coated (354234, Corning, USA) in advance. Then, the cells were incubated at 37 °C for 24-48 h to allow for migration or invasion. For quantification, the cells were fixed with 4% paraformaldehyde for 15 min, stained with hematoxylin for 10 min at room temperature, and counted in four randomly chosen fields (× 200) under a microscope.

**Tube formation on Matrigel**

A total of 150 µl Matrigel (354248, Corning) solution per well was coated on a 48-well plate allowed to solidify at 37ºC for 1 h. Human umbilical vein endothelial cells (HUVECs) suspended in different CMs were seeded on the Matrigel cushion at the density of (4 × 10^4^) cells/well, and then incubated for 2 h. Kinetics of tube formation at different time points was observed and counted.

**RNA isolation and** **RT-qPCR**

Total RNA was isolated from cell lines or tissues using the TRIzol reagent (TaKaRa, China). RT-qPCR was performed using the PrimeScript RT Reagent Kit (#RR035A, TaKaRa, China) and SYBR Premix Ex Taq (#RR820A, TaKaRa, Dalian, China) according to the manufacturer’s instructions. The specific primers used are listed in Supplementary Table 1. The RT-qPCR results were analyzed to obtain the Ct values of the amplified products, and data were analyzed by the 2-ΔΔCt method.

**Western blotting**

Western blotting was conducted as previously described [12, 13]. Protein lysates were prepared, subjected to 10% sodium dodecyl sulfate polyacrylamide gel electrophoresis (SDS-PAGE), transferred onto polyvinylidene difluoride (PVDF) membranes and blotted according to standard methods using the following antibodies: RUNX1 (1:4000, 25315-1-AP, Proteintech), cyclin D1 (1:1000, #2978, CST), LEF1 (1:1000, #2230, CST), c-Myc (1:1000, #5605, CST), PTCH1 (1:1000, #2468, CST), PTCH2 (1:1000, #2470, CST), GLI1 (1:1000, #3538, CST), SUFU (1:1000, #2520, CST), Shh (1:1000, #2207, CST), PCNA (1:10000, 10205-2-AP, Proteintech) and GAPDH (1:80000, 60004-1-Ig, Proteintech). When CRC cells were stimulated with THP-1 derived CMs, cell lysates were prepared using a commercial Nuclear-Cytosol Extraction Kit (FD0199), and subsequent steps were performed.

**Enzyme-linked immunosorbent assay (ELISA)**

To detect the levels of IL-10 and PDGF-BB secreted by THP-1 treated with CRC cells-derived CMs, the adhered THP-1 was first treated with different CMs for 24 h, then washed twice with PBS, and cultured in fresh DMEM complete medium for another 24 h. The supernatant of each group was finally collected for detection. The expression of soluble PDGF-BB, IL-10 and CCL2 in the cell supernatant was detected by the commercial ELISA kit, according to the manufacturer’s instructions. Positive controls were supplied in the kit.

**Human Protein Atlas Analysis (HPA)**

HPA is a comprehensive website (http://www.Proteinatlas.org/) using antibody-based imaging, mass spectrometry-based proteomics, transcriptomics and systems biology to map human protein in cells, tissues and organs. In the present study, we analyzed the expression of RUNX1 in CRC and normal tissues using the immunohistochemistry results in HPA.

**Oncomine analysis and The Cancer Genome Atlas (TCGA) Cancer Dataset**

The Oncomine database (<http://www.oncomine.org/resource/login.html>) compiles 715 datasets and 86733 samples. We applied this database to assess RUNX1 expression profile in various tumors. In addition to the above databases, a dataset of RNA-seq expression profiles were downloaded from the University of California, Santa Cruz (UCSC) Cancer Genome Browser (https://xenabrowser.net/datapages/?cohort=TCGA%20Pan-Cancer%20(PANCAN)&removeHub= https%3A%2F%2Fxena.treehouse.gi.ucsc.edu%3A443). All figures from this database (TCGA) in our study were generated in R v3.6.0.

**Gene Expression Profiling Interaction Analysis (GEPIA)**

GEPIA is a multifunctional website (http://gepia.cancer-pku.cn/) based on the big genomic data (RNA sequencing data of 9736 tumors and 8587 normal samples) in The Cancer Genome Atlas (TCGA) and The Genotype-Tissue Expression (GTEx) databases. GEPIA performs function of seven sections: General, Differential Genes, Expression DIY, Survival, Similar Genes, Correlation and Principal Component Analysis. In the present study, we used the “Expression DIY” and “Correlation” function of GEPIA database to analyze the expression levels of RUNX1 in CRC and correlation between RUNX1 and immune-related molecules. To explore the association between RUNX1 and endothelial markers, the *P* values and correlation coefficients between these genes were first obtained from the GEPIA2 website. Then, the above parameters were used to plot correlation bubbles in EXCEL.

**Tumor Immune Estimation Resource Database Analysis (TIMER)**

TIMER (http://cistrome.shinyapps.io/timer/) is a web server for analysis of immune infiltrates in different cancers. This database could estimate the abundances of six immune infiltrates (B cells, CD8+ T cells, CD4+ T cells, macrophages, neutrophils and dendritic cells) using TIMER algorithm. Moreover, this website also offers “Diff Exp” module to explore gene expressions between diverse tumor and normal tissues. In our study, we employed this database to assess RUNX1 expression in different tumor and normal tissues. We further explored the correlation between RUNX1 expression and immune cell infiltration/immune infiltrating cell markers in CRC.

**Supplementary Table 1 Primer sequences** **used for real-time PCR (5' to 3')**

| Gene | Forward primer | Reverse primer |
| --- | --- | --- |
| RUNX1  CCL2  CD163  Arg1  CD206  IL-10  GAPDH | CACTGTGATGGCTGGCAATGATG  AAGAAGCTGTAGTATTTGTCACCAAGCTCA  GGTGGACACAGAATGGTTCTTC  GACCTGCCCTTTGCTGACATCC  TCCGACCCTTCCTTGACTAATCCTC  GGGTTGCCAAGCCTTGTCTGAG  GCACCGTCAAGGCTGAGAAC | CTCTGTGGTAGGTGGCGACTTG  CATCAGGTACGATCCAGGCT  CCAGGAGCGTTAGTGACAGC  TCTTCTTGACTTCTGCCACCTTGC  AGTATGTCTCCGCTTCATGCCATTG  CCTTGATGTCTGGGTCTTGGTTCTC  TGGTGAAGACGCCAGTGGA |

| **Supplementary Table 2 Correlation between RUNX1 and immune cell markers** | | | | | | | | | | | | | | | | |
| --- | --- | --- | --- | --- | --- | --- | --- | --- | --- | --- | --- | --- | --- | --- | --- | --- |
| Description | | | Gene markers | COAD | | | | | READ | | | | COAD.READ | | | |
|  |  |  |  | Tumor | | | Normal | | Tumor | | Normal | | Tumor | | Normal | |
|  |  |  |  | R | | *P* | R | *P* | R | *P* | R | *P* | R | *P* | R | *P* |
| Monocyte | | | CD86 | 0.45 | | 6.90E-15 | 0.15 | 0.34 | 0.59 | 6.30E-10 | -0.39 | 0.26 | 0.48 | 7.20E-23 | -0.0005 | 1 |
|  | | | CD115(CSF1R) | 0.53 | | 1.50E-21 | 0.29 | 0.07 | 0.57 | 2.30E-09 | -0.079 | 0.84 | 0.54 | 1.50E-29 | 0.24 | 0.087 |
| TAM | | | CCL2 | 0.47 | | 9.00E-17 | 0.041 | 0.8 | 0.45 | 5.20E-06 | -0.042 | 0.92 | 0.47 | 5.90E-22 | -0.053 | 0.71 |
|  | | | CD68 | 0.43 | | 5.30E-14 | 0.17 | 0.28 | 0.5 | 3.60E-07 | -0.48 | 0.17 | 0.45 | 8.10E-20 | 0.092 | 0.52 |
|  | | | IL10 | 0.47 | | 2.50E-16 | 0.1 | 0.52 | 0.49 | 5.60E-07 | -0.64 | 0.054 | 0.48 | 3.70E-22 | -0.055 | 0.7 |
| M1 Macrophage | | | INOS(NOS2） | -0.14 | | 0.021 | 0.38 | 0.016 | 0.064 | 0.54 | 0.48 | 0.17 | -0.089 | 0.089 | 0.41 | 0.0032 |
|  |  |  | IRF5 | 0.22 | | 0.00021 | 0.2 | 0.21 | 0.18 | 0.092 | 0.67 | 0.039 | 0.21 | 6.00E-05 | 0.25 | 0.081 |
|  |  |  | COX2(PTGS2) | 0.24 | | 6.30E-05 | 0.015 | 0.93 | 0.5 | 3.30E-07 | -0.042 | 0.92 | 0.31 | 1.00E-09 | -0.036 | 0.8 |
| M2 Macrophage | | | CD163 | 0.46 | | **8.10E-16** | 0.25 | 0.12 | 0.5 | **3.00E-07** | 0.079 | 0.84 | 0.47 | **8.70E-22** | 0.23 | 0.1 |
|  |  |  | VSIG4 | 0.45 | | **3.20E-15** | -0.013 | 0.93 | 0.49 | **8.50E-07** | -0.76 | 0.016 | 0.46 | **9.90E-21** | -0.15 | 0.3 |
|  |  |  | MS4A4A | 0.45 | | **2.70E-15** | 0.11 | 0.47 | 0.55 | **1.10E-08** | -0.64 | 0.054 | 0.48 | **1.30E-22** | -0.028 | 0.85 |
| B cell | | | CD19 | 0.22 | | 0.00031 | 0.18 | 0.26 | 0.24 | 0.019 | 0.5 | 0.14 | 0.22 | 2.60E-05 | 0.21 | 0.14 |
|  | | | CD79A | 0.31 | | 1.20E-07 | 0.29 | 0.064 | 0.2 | 0.059 | 0.43 | 0.22 | 0.28 | 5.50E-08 | 0.31 | 0.026 |
| CD8+T cell | | | CD8A | 0.24 | | 5.70E-05 | -0.013 | 0.93 | 0.32 | 0.0019 | 0.59 | 0.08 | 0.26 | 6.50E-07 | 0.069 | 0.63 |
|  | | | CD8B | 0.14 | | 0.02 | -0.25 | 0.12 | 0.13 | 0.22 | 0.21 | 0.56 | 0.13 | 0.01 | -0.17 | 0.24 |
| CD4+T cell | | | CD4 | 0.5 | | 1.30E-18 | 0.34 | 0.029 | 0.51 | 1.60E-07 | 0.33 | 0.35 | 0.51 | 2.20E-25 | 0.31 | 0.026 |
|  | | | CD40LG(CD40L) | 0.35 | | 2.80E-09 | 0.16 | 0.31 | 0.22 | 0.036 | 0.31 | 0.39 | 0.31 | 6.90E-10 | 0.17 | 0.23 |
|  | | | CXCR4 | 0.47 | | 1.20E-16 | 0.25 | 0.11 | 0.54 | 3.50E-08 | 0.5 | 0.14 | 0.49 | 4.20E-23 | 0.25 | 0.075 |
| T cell  (general) | | | CD3D | 0.22 | | 0.00025 | -0.003 | 0.99 | 0.24 | 0.024 | 0.12 | 0.76 | 0.22 | 2.50E-05 | 0.028 | 0.85 |
|  |  |  | CD3E | 0.33 | | 1.40E-08 | 0.21 | 0.2 | 0.32 | 0.0018 | 0.62 | 0.06 | 0.33 | 1.30E-10 | 0.27 | 0.055 |
|  | | | CD28 | 0.44 | | 2.50E-14 | 0.33 | 0.038 | 0.53 | 4.90E-08 | 0.37 | 0.3 | 0.46 | 5.60E-21 | 0.28 | 0.048 |
|  |  |  | CD2 | 0.32 | | 4.10E-08 | 0.12 | 0.47 | 0.34 | 0.00098 | 0.41 | 0.25 | 0.33 | 1.60E-10 | 0.15 | 0.29 |
| Neutrophil | | CD11b(ITGAM) | | 0.54 | | 2.10E-22 | 0.24 | 0.13 | 0.57 | 2.20E-09 | 0.067 | 0.86 | 0.55 | 9.40E-31 | 0.19 | 0.19 |
|  |  | CCR7 | | 0.34 | | 4.70E-09 | 0.21 | 0.18 | 0.41 | 5.70E-05 | 0.61 | 0.066 | 0.36 | 1.10E-12 | 0.21 | 0.13 |
|  | CD66 b(CEACAM8) | | | | -0.22 | 0.00021 | 0.085 | 0.6 | -0.13 | 0.23 | 0.67 | 0.035 | -0.2 | 0.00015 | 0.21 | 0.14 |
| Natural killer cell | | | KIR2DL1 | 0.1 | | 0.083 | 0.45 | 0.0034 | 0.29 | 0.0046 | -0.59 | 0.075 | 0.15 | 0.0045 | 0.29 | 0.039 |
|  |  |  | KIR2DL3 | 0.16 | | 0.0062 | -0.28 | 0.074 | 0.068 | 0.52 | -0.51 | 0.14 | 0.14 | 0.0076 | -0.24 | 0.091 |
|  | | | KIR2DL4 | 0.054 | | 0.37 | -0.19 | 0.24 | 0.049 | 0.64 | 0.64 | 0.047 | 0.052 | 0.32 | -0.027 | 0.85 |
|  | | | KIR3DL1 | 0.095 | | 0.12 | -0.063 | 0.7 | 0.12 | 0.25 | 0.11 | 0.76 | 0.1 | 0.056 | -0.038 | 0.79 |
|  | | | KIR3DL2 | 0.22 | | 2.00E-04 | 0.19 | 0.23 | 0.19 | 0.067 | 0.44 | 0.2 | 0.21 | 4.40E-05 | 0.24 | 0.095 |
|  | | | KIR3DL3 | 0.069 | | 0.25 | 0.27 | 0.082 | 0.11 | 0.31 | 0.51 | 0.13 | 0.078 | 0.14 | 0.31 | 0.026 |
|  | | | KIR2DS4 | 0.18 | | 0.0028 | 0.35 | 0.023 | 0.097 | 0.36 | -0.0075 | 0.98 | 0.16 | 0.0024 | 0.29 | 0.04 |
|  | | | NCAM1(CD56) | 0.48 | | 5.90E-17 | 0.09 | 0.58 | 0.18 | 0.084 | 0.24 | 0.51 | 0.41 | 2.10E-16 | 0.072 | 0.61 |
|  | | | FCGR3A(CD16) | 0.45 | | 3.80E-15 | -0.052 | 0.75 | 0.58 | 1.80E-09 | -0.21 | 0.56 | 0.48 | 7.20E-23 | -0.053 | 0.71 |
| Dendritic cell | | | HLA-DPB1 | 0.41 | | 1.60E-12 | 0.078 | 0.63 | 0.43 | 1.70E-05 | 0.13 | 0.73 | 0.41 | 1.10E-16 | 0.064 | 0.66 |
|  |  |  | HLA-DQB1 | 0.22 | | 0.00032 | 0.11 | 0.49 | 0.19 | 0.072 | 0.2 | 0.58 | 0.2 | 7.70E-05 | 0.13 | 0.37 |
|  | | | HLA-DRA | 0.32 | | 7.90E-08 | -0.093 | 0.56 | 0.36 | 0.00041 | -0.38 | 0.28 | 0.33 | 1.30E-10 | -0.18 | 0.22 |
|  | | | HLA-DPA1 | 0.38 | | 1.00E-10 | 0.02 | 0.9 | 0.41 | 5.70E-05 | -0.055 | 0.89 | 0.39 | 1.30E-14 | 0.024 | 0.87 |
|  | | | BCDA-1(CD1C) | 0.37 | | 1.40E-10 | -0.2 | 0.2 | 0.2 | 0.057 | 0.42 | 0.23 | 0.33 | 1.30E-10 | -0.21 | 0.14 |
|  | | | BDCA-4(NRP1) | 0.58 | | 4.20E-26 | 0.39 | 0.012 | 0.65 | 1.80E-12 | 0.055 | 0.89 | 0.6 | 2.00E-37 | 0.31 | 0.028 |
|  | | | CD11c(ITGAX) | 0.51 | | 1.50E-19 | 0.37 | 0.018 | 0.65 | 2.40E-12 | 0.5 | 0.14 | 0.54 | 1.00E-29 | 0.36 | 0.0086 |
| Th1 | | | T-bet(TBX21) | 0.32 | | 5.80E-08 | 0.24 | 0.14 | 0.4 | 6.40E-05 | 0.54 | 0.11 | 0.34 | 3.90E-11 | 0.31 | 0.026 |
|  | | | STAT4 | 0.38 | | 5.20E-11 | 0.17 | 0.28 | 0.43 | 2.20E-05 | 0.3 | 0.4 | 0.39 | 5.00E-15 | 0.19 | 0.18 |
|  | | | STAT1 | 0.36 | | 6.70E-10 | 0.27 | 0.083 | 0.5 | 3.10E-07 | -0.14 | 0.71 | 0.39 | 5.00E-15 | 0.23 | 0.11 |
|  | | | IFN-γ(IFNG) | 0.15 | | 0.012 | 0.044 | 0.79 | 0.3 | 0.0039 | -0.7 | 0.025 | 0.19 | 0.00036 | -0.041 | 0.77 |
|  | | | TNF-α(TNF) | 0.33 | | 2.20E-08 | 0.13 | 0.4 | 0.41 | 5.10E-05 | 0.29 | 0.41 | 0.35 | 5.00E-12 | 0.13 | 0.38 |
| Th2 | | | GATA3 | 0.46 | | 5.40E-16 | 0.34 | 0.029 | 0.47 | 2.20E-06 | 0.7 | 0.024 | 0.47 | 3.90E-21 | 0.4 | 0.0038 |
|  | | | STAT6 | 0.26 | | 1.20E-05 | 0.016 | 0.92 | 0.32 | 0.002 | 0.47 | 0.18 | 0.27 | 1.10E-07 | 0.12 | 0.42 |
|  | | | STAT5A | 0.44 | | 1.50E-14 | 0.35 | 0.025 | 0.25 | 0.019 | 0.33 | 0.35 | 0.39 | 5.70E-15 | 0.34 | 0.015 |
|  | | | IL13 | 0.26 | | 1.80E-05 | 0.012 | 0.94 | 0.2 | 0.052 | 0.41 | 0.24 | 0.24 | 3.10E-06 | 0.08 | 0.58 |
| Tfh | | | BCL6 | 0.58 | | 5.80E-26 | 0.28 | 0.078 | 0.53 | 5.20E-06 | 0.33 | 0.35 | 0.56 | 3.50E-32 | 0.24 | 0.09 |
|  | | | IL21 | 0.18 | | 0.0034 | 0.51 | 0.00073 | 0.24 | 0.019 | 0.51 | 0.14 | 0.19 | 2.00E-04 | 0.46 | 0.00066 |
| Th17 | | | STAT3 | 0.43 | | 5.70E-14 | 0.54 | 0.00034 | 0.51 | 2.70E-07 | 0.48 | 0.17 | 0.45 | 1.90E-19 | 0.55 | 4.60E-05 |
|  | | | IL17A | -0.097 | | 0.11 | 0.069 | 0.67 | -0.065 | 0.54 | -0.17 | 0.64 | -0.086 | 0.1 | 0.02 | 0.89 |
| Treg | | | FOXP3 | 0.43 | | 8.70E-14 | 0.32 | 0.045 | 0.48 | 1.40E-06 | 0.67 | 0.039 | 0.44 | 4.70E-19 | 0.37 | 0.0083 |
|  | | | CCR8 | 0.47 | | 1.30E-16 | 0.39 | 0.012 | 0.5 | 3.90E-07 | 0.41 | 0.25 | 0.48 | 2.10E-22 | 0.34 | 0.014 |
|  | | | STAT5B | 0.44 | | 1.50E-14 | 0.21 | 0.2 | 0.41 | 5.40E-05 | 0.19 | 0.61 | 0.44 | 1.40E-18 | 0.15 | 0.28 |
|  | | | TGFβ(TGFB1) | 0.52 | | 3.00E-20 | 0.36 | 0.022 | 0.6 | 3.00E-10 | 0.42 | 0.23 | 0.54 | 1.30E-28 | 0.36 | 0.01 |
| T cell exhaustion | | | PD-1(PDCD1) | 0.3 | | 4.50E-07 | 0.31 | 0.05 | 0.48 | 1.10E-06 | 0.62 | 0.06 | 0.33 | 7.10E-11 | 0.32 | 0.02 |
|  |  |  | CTLA4 | 0.4 | | 6.60E-12 | 0.24 | 0.13 | 0.48 | 1.20E-06 | 0.44 | 0.2 | 0.42 | 7.80E-17 | 0.25 | 0.079 |
|  | | | LAG3 | 0.23 | | 0.00014 | 0.2 | 0.22 | 0.35 | 0.00063 | 0.54 | 0.11 | 0.25 | 1.40E-06 | 0.25 | 0.076 |
|  | | | TIM-3(HAVCR2) | 0.46 | | 1.80E-15 | 0.14 | 0.38 | 0.55 | 1.10E-08 | -0.39 | 0.26 | 0.48 | 1.80E-22 | -0.0038 | 0.98 |
|  | | | GZMB | 0.068 | | 0.26 | 0.18 | 0.26 | 0.047 | 0.66 | -0.1 | 0.79 | 0.06 | 0.25 | 0.13 | 0.35 |
|  | | | BTLA | 0.27 | | 4.40E-06 | 0.13 | 0.43 | 0.22 | 0.038 | 0.03 | 0.93 | 0.26 | 4.40E-07 | 0.11 | 0.44 |
|  | | | CD244(SLAMF4) | 0.26 | | 1.50E-05 | -0.071 | 0.66 | 0.24 | 0.021 | -0.079 | 0.84 | 0.25 | 1.20E-06 | -0.061 | 0.67 |
|  | | | CD274(PD-L1) | 0.31 | | 1.50E-07 | 0.29 | 0.066 | 0.43 | 1.50E-05 | 0.5 | 0.14 | 0.34 | 2.30E-11 | 0.28 | 0.047 |
|  | | | CD96 | 0.33 | | 2.20E-08 | 0.029 | 0.86 | 0.37 | 0.00024 | 0.3 | 0.41 | 0.34 | 3.10E-11 | 0.075 | 0.6 |
|  | | | IDO1 | 0.24 | | 6.90E-05 | 0.25 | 0.11 | 0.31 | 0.0025 | 0.1 | 0.79 | 0.25 | 9.10E-07 | 0.19 | 0.18 |
|  | | | KDR | 0.56 | | 8.10E-24 | 0.52 | 0.00043 | 0.54 | 2.30E-08 | 0.018 | 0.97 | 0.56 | 2.70E-31 | 0.47 | 0.00056 |
|  | PDCD1LG2(PD-L2) | | | 0.49 | | 2.60E-18 | 0.11 | 0.5 | 0.57 | 4.30E-09 | -0.43 | 0.21 | 0.51 | 4.40E-26 | -0.0071 | 0.96 |
|  | | | TGFBR1 | 0.58 | | 8.80E-26 | 0.31 | 0.046 | 0.7 | 1.10E-14 | 0.055 | 0.89 | 0.61 | 1.80E-38 | 0.23 | 0.11 |
|  | | | TIGIT | 0.36 | | 4.70E-10 | 0.25 | 0.11 | 0.41 | 6.10E-05 | 0.52 | 0.13 | 0.37 | 2.10E-13 | 0.31 | 0.028 |

| **Supplementary Table 3 Correlation between Runx1 and macrophage markers** | | | | | | | | | |
| --- | --- | --- | --- | --- | --- | --- | --- | --- | --- |
| Description | Cene markers | COAD (n=457) | | | | READ (n=166) | | | |
|  |  | None | | Purity | | None | | Purity | |
|  |  | Cor | *P* | Cor | *P* | Cor | *P* | Cor | *P* |
| Monocyte | CD86 | 0.339 | 1.24E-13 | 0.287 | 4.04E-09 | 0.337 | 1.05E-05 | 0.289 | 5.50E-04 |
|  | CD115(CSF1R) | 0.39 | 0.00E+00 | 0.335 | 4.06E-12 | 0.362 | 1.94E-06 | 0.315 | 1.62E-04 |
| TAM | CCL2 | 0.366 | 5.65E-16 | 0.307 | 2.56E-10 | 0.319 | 3.15E-05 | 0.261 | 1.92E-03 |
|  | CD68 | 0.305 | 2.75E-11 | 0.258 | 1.38E-07 | 0.289 | 1.66E-04 | 0.254 | 2.51E-03 |
|  | IL10 | 0.278 | 1.33E-09 | 0.233 | 2.05E-06 | 0.262 | 6.46E-04 | 0.211 | 1.28E-02 |
| M1 Macrophage | INOS(NOS2） | -0.135 | 3.80E-03 | -0.169 | 6.05E-04 | 0.051 | 5.12E-01 | 0.023 | 7.92E-01 |
|  | IRF5 | 0.156 | 8.39E-04 | 0.174 | 4.24E-04 | 0.02 | 8.02E-01 | 0.011 | 8.95E-01 |
|  | COX2(PTGS2) | 0.214 | 3.66E-06 | 0.168 | 6.70E-04 | 0.365 | 1.58E-06 | 0.333 | 6.16E-05 |
| M2 Macrophage | CD163 | 0.396 | 0.00E+00 | 0.346 | 7.13E-13 | 0.408 | 6.34E-08 | 0.368 | 8.17E-06 |
|  | VSIG4 | 0.301 | 5.87E-11 | 0.231 | 2.47E-06 | 0.212 | 6.28E-03 | 0.181 | 3.31E-02 |
|  | MS4A4A | 0.301 | 6.04E-11 | 0.242 | 8.16E-07 | 0.298 | 1.03E-04 | 0.257 | 2.28E-03 |

**Supplementary Table 4 Abbreviations**

| abbreviation |  | abbreviation |  |
| --- | --- | --- | --- |
| BSA  CAM  CCL2  CDH5  ChIP  CMs  COAD  CRC  ELISA  EMT  FGF  GEPIA  GLI1  GM-CSF  GSEA  H&E  HPI  HUVECs  ICAMs  IF  IFN-γ  IHC  KDR | bovine serum albumin  chick embryo chorioallantoic membrane  chemokine 2  cadherin 5  Chromatin immunoprecipitation  conditioned mediums  colon adenocarcin  coloractal cancer  enzyme-linked immunosorbent assay  epithelial to mesenchymal transition  fibroblast growth factor  Gene Expression Profiling Interaction Analysis  glioma-associated oncogene homolog 1  granulocyte macrophage colony stimulating factor  Gene set enrichment analysis  hemogenic endothelium  hedgehog pathway inhibitor  human umbilical vein endothelial cells  intercellular adhesion molecules  immunofluorescence  interferon-γ  immunohistochemistry  Kinase insert domain receptor | LEF1  PBS  PDGF  PECAM-1  PMA  PTCH  PVDF  qRT-PCR  RUNX1  SELE  SELP  SDS-PAGE  Shh  SPF  SUFU  TAMs  TCGA  TIMER  TLR  TME  VCAM-1  VEGF | lymphoid enhancer-binding factor 1  phosphate-buffered saline  platelet-derived growth factor  platelet endothelial cell adhesion molecule  phorbol 12-myristate 13-acetate  patched receptor  polyvinylidene difluoride  quantitative real-time polymerase chain reaction  runt-related transcription factor 1  E-selectin  P-selectin  sodium dodecyl sulfate polyacrylamide gel electrophoresis  sonic Hh  specific-pathogen-free  suppressor of fused  tumor associated macrophages  the cancer genome atlas  Tumor Immune Estimation Resource Database Analysis  Toll like receptor  tumor microenvironment  vascular cell adhesion molecules  vascular endothelial growth factor |
